# Multi‐Tissue Genetic Regulation of RNA Editing in Pigs

**DOI:** 10.1002/advs.202518238

**Published:** 2026-01-18

**Authors:** Xiangchun Pan, Wentao Gong, Xiaodian Cai, Jinyan Teng, Jiali Cai, Haonan Zeng, Wondossen Ayalew, Qingpeng Shen, Zhanming Zhong, Yifei Wang, Wenjing Zhang, Yuhan Tian, Dantong Xu, Yahui Gao, Hongwei Yin, Yuebo Zhang, Jiahui Hou, Tianru Zhou, Jiaqi Li, Lingzhao Fang, Xiaolong Yuan, Zhe Zhang

**Affiliations:** ^1^ State Key Laboratory of Swine and Poultry Breeding Industry Guangdong Laboratory for Lingnan Modern Agriculture Guangdong Provincial Key Lab of Agro‐Animal Genomics and Molecular Breeding College of Animal Science South China Agricultural University Guangzhou China; ^2^ Shenzhen Branch Guangdong Laboratory for Lingnan Modern Agriculture Agricultural Genomics Institute at Shenzhen Genome Analysis Laboratory of the Ministry of Agriculture Chinese Academy of Agricultural Sciences Shenzhen China; ^3^ College of Animal Science and Technology Hunan Agricultural University Changsha China; ^4^ Center for Quantitative Genetics and Genomics (QGG) Aarhus University Aarhus Denmark

**Keywords:** PigGTEx, RNA editing, RNA editing quantitative trait loci, RNA‐seq, SNP

## Abstract

RNA editing introduces transcriptomic variation and may contribute to complex trait regulation. However, its genetic determination in pigs remains unclear. Here, using 5457 PigGTEx RNA‐seq samples across 34 porcine tissues, we construct a comprehensive RNA editome and map 49 614 cis‐editing quantitative trait loci (edQTLs). These edQTLs define a distinct regulatory layer, with over 32% acting independently of expression or splicing QTLs (eQTLs/sQTLs). Crucially, edQTLs are significantly enriched in GWAS loci for 33 complex traits and show a superior contribution to heritability over eQTLs/sQTLs in some traits like growth and reproduction. Colocalization analyses pinpoint diverse causal mechanisms, revealing both edQTL‐specific effects (e.g., *ERCC5*) and coordinated events where single variants pleiotropically modulate RNA editing, splicing, and gene expression. Human‐pig comparative analysis identifies hundreds of evolutionarily conserved editing events linked to fundamental neurological pathways. This study provides the first comprehensive multi‐tissue porcine RNA editome and edQTL map, establishing RNA editing as a key mechanism linking genetic variation to phenotypic diversity in a major agricultural species and offering a valuable resource for future functional genomics research.

## Graphical abstract

This study presents the first multi‐tissue map of RNA editing and its genetic regulation in pigs. By integrating RNA editing profiles, edQTL mapping, GWAS, and cross‐species comparisons, this work establishes RNA editing as a distinct regulatory layer linking genetic variation to complex traits, highlighting its functional and evolutionary significance.

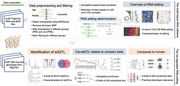

## 1 Introduction

RNA editing is a crucial cellular process wherein the sequence is altered at the RNA level without modifying the corresponding genomic sequences [1, 2, 3]. The well‐studied types of substitutional RNA editing include the conversion of adenosine to inosine (A‐to‐I) and cytosine to uracil (C‐to‐U) in mammals [4, 5]. The A‐to‐I RNA editing is catalyzed by enzymes of the adenosine deaminases acting on RNA (ADAR) members, which include *ADAR1* (also known as *ADAR*), *ADAR2* (also known as *ADARB1*), and *ADAR3* (also known as *ADARB2*) [6, 7]. The process of A‐to‐I editing can significantly influence splicing, RNA structure, translation, and the function of proteins [8]. In contrast, the C‐to‐U RNA editing is a complex process that encompasses the participation of editing enzymes from the APOBECs (*APOBEC1*, *APOBEC2*, *APOBEC3B*), as well as the requirement for cofactors like ACF and RBM47 that facilitate its binding to the substrate [7, 9]. Similarly, C‐to‐U editing is instrumental in multiple biological processes, encompassing gene expression regulation, mRNA stability, and the diversification of protein isoforms [9, 10]. However, the regulatory mechanisms of RNA editing, particularly tissue‐specific enzyme contributions and their impact on editing efficiency, remain poorly characterized in pigs.

Beyond enzyme‐mediated regulation, the contribution of genetic variation to RNA editing has garnered increasing attention [11]. At the population level, molecular quantitative trait loci (molQTL) studies adeptly bridge Genome‐Wide Association Study (GWAS) variants to genetic mechanisms, delving into the genetic effects on molecular traits [12]. For instance, gene expression QTL (eQTL) and alternative splicing QTL (sQTL) have been used to successfully study the regulatory architecture of gene expression and gene alternative splicing in both humans and livestock [13, 14, 15, 16, 17, 18, 19]. In addition to these, other molecular traits have been studied using the molQTL approach, such as DNA methylation (meQTL) [20], protein abundance (pQTL) [21], and RNA editing (edQTL) [22]. The edQTL are DNA variants that modulate the RNA editing level at a specific site, and researchers are delving into these variants to uncover how they influence the landscape of RNA editing [11, 23, 24, 25]. Additionally, edQTL studies have unveiled the possibility that RNA editing variability can influence complex traits [22, 23, 24, 25]. For example, Li et al. discovered that dsRNA editing is a common inflammatory disease mechanism by edQTL analysis [22]. The molQTL studies in pigs have successfully identified five major regulatory categories—encompassing *cis*‐eQTLs for coding genes, *cis*‐sQTLs for alternative splicing, *cis*‐eeQTLs for exons, *cis*‐lncQTLs for long non‐coding RNAs, and *cis*‐enQTLs for enhancers—elucidating the regulatory mechanisms behind the majority of 207 complex traits through an extensive analysis of 5457 unique RNA‐seq samples in the pilot phase of the Pig Genotype‐Tissue Expression (PigGTEx) project [16]. Nevertheless, the role of edQTLs in modulating RNA editing across tissues and their potential impact on complex traits remains largely unexplored in pigs. It is of significance to deeply understand the role of RNA editing in the manifestation of prevalent and complex traits in pigs.

**FIGURE 1:**
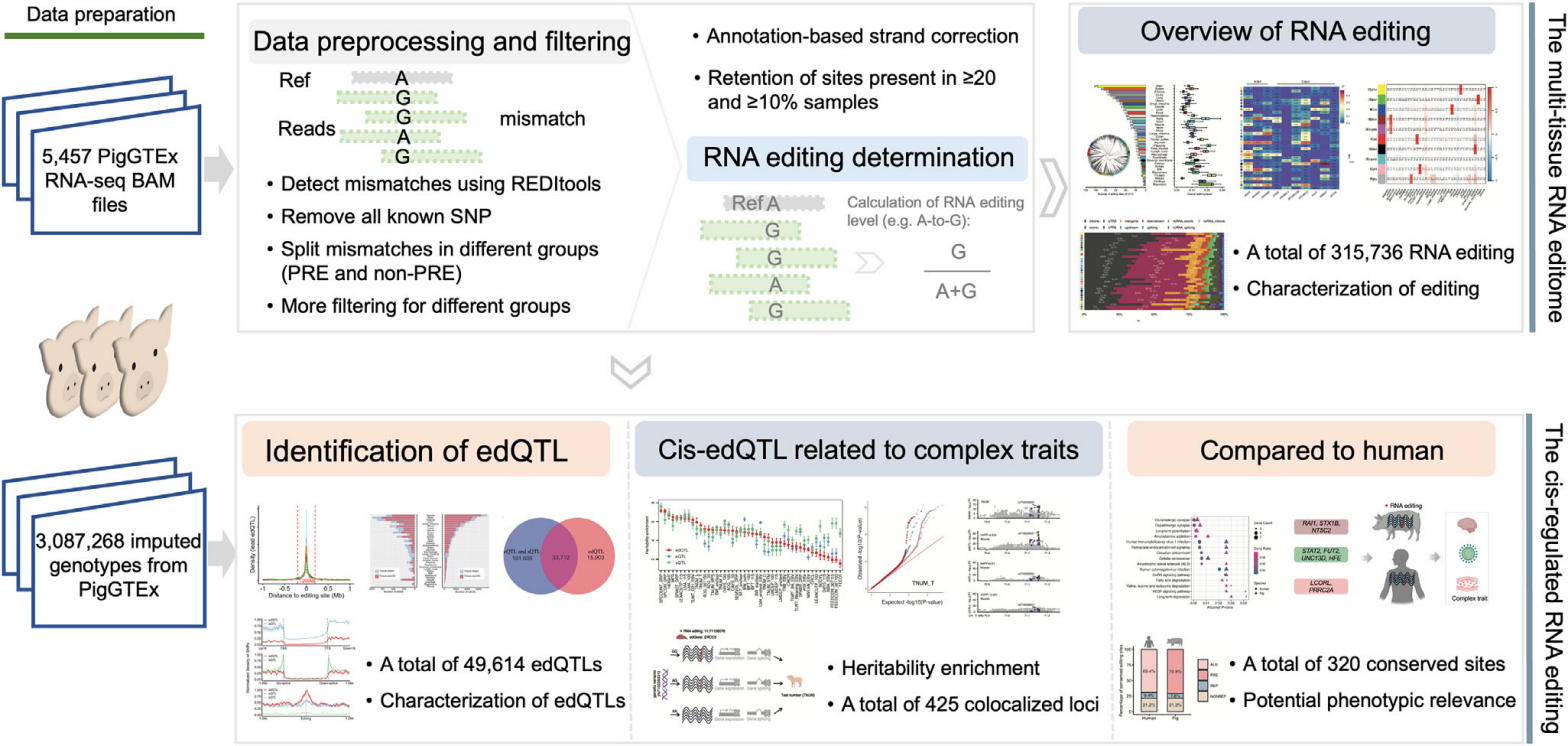
Overview of the study design and analytical framework. RNA‐seq data from PigGTEx are processed through a stringent multi‐step pipeline to identify high‐confidence RNA editing sites. All RNA editing levels are calculated as the ratio of mismatched reads to total coverage at each site. A comprehensive annotation of RNA editing profiles is performed across 34 pig tissues. Subsequent analyses focus on the genetic underpinnings of RNA editing, which identified editing QTLs (edQTL) in multiple tissues of pigs using the imputed genotypes from PigGTEx. It then examines the links between these edqtls and phenotypic traits, drawing parallels and distinctions with splicing QTLs (sQTLs) and expression QTLs (eQTLs), thereby shedding light on the crucial genetic role of RNA editing. Finally, a comparative analysis with human profiles revealed evolutionarily conserved regulatory features.

Here, we sought to unveil the global atlas and the principal genetic drivers of RNA editing across diverse tissues in pigs by applying a multi‐step workflow (Figure 1). To investigate the occurrence of RNA editing in pigs and its potential genetic underpinnings, we first identified RNA editing across 34 tissues. Further, we characterized the porcine RNA editome utilizing data from PigGTEx sources (https://piggtex.farmgtex.org/). Subsequently, we primarily conducted comprehensive edQTL analysis by integrating eQTL, sQTL, and GWAS to determine the functional contribution of edQTLs. We further performed a small‐scale comparison with humans to assess the conservation of RNA editing sites and regulatory features. Our findings provide important insights into the genetic architecture of RNA editing and its potential impact on complex traits across multiple porcine tissues. All edQTL data are freely available at the PigGTEx portal.

## 2 Results

### 2.1 RNA Editing Profile Across 34 Tissues in Pigs

**FIGURE 2:**
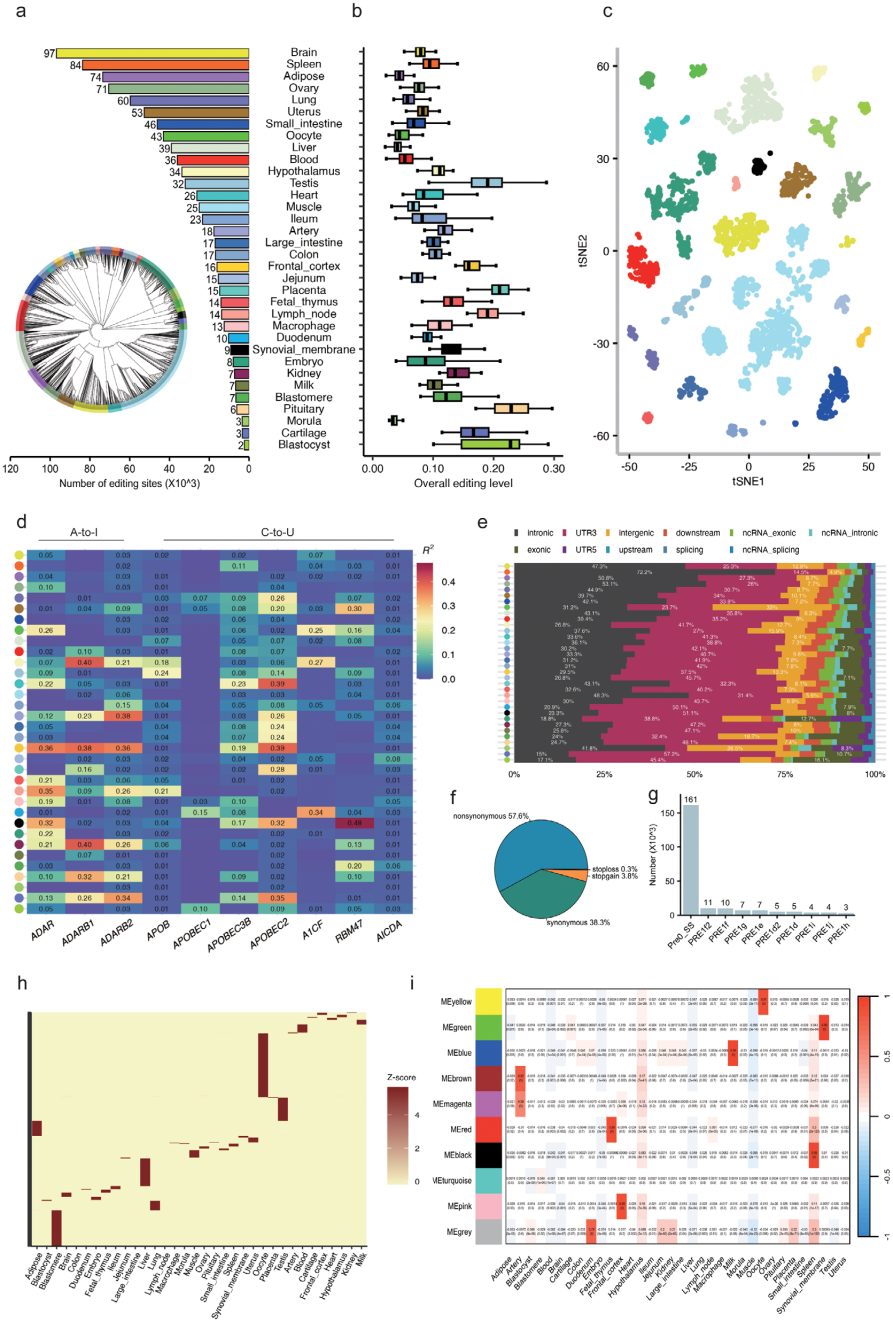
Overall RNA editing profiles in pigs. (a) Number of RNA editing sites in each tissue, and clustering of 5457 RNA‐seq samples based on the RNA editing level (bottom left). Color codes are consistent across all tissues presented in the article. (b) The overall editing level in each 34 tissues, where each box plot corresponds to samples from a specific tissue type. (c) The editing pattern using t‐SNE dimensionality‐reduction analysis. (d) Correlations between expression levels of RNA editing enzymes/relevance‐factors and overall editing level for A‐to‐I (A‐to‐G) and C‐to‐U (C‐to‐T), respectively. (e) Distribution characteristics of RNA‐edited genomic regions in different tissues, with only the top three proportions displayed in each tissue. (f) Proportion of exonic variant functions. (g) Proportions of RNA editing sites within different elements. (h) Tissue‐specific RNA editing. The editing levels are normalized across samples for each site. (i) Co‐editing network analysis of 1677 sites that exhibited high variation across tissues (coefficient of variance > 0.8), constructed using per‐site editing levels averaged across samples within each tissue.

To identify RNA editing sites in pigs and establish a comprehensive editing map, we applied stringent quality control measures to all 5457 RNA‐seq data (Figure S1A and “Methods”). After removing mismatches explained by systematic alignment or sequencing artifacts, we identified 315 736 unique RNA editing sites across all RNA‐seq samples from 34 tissues, predominantly composed of 237 674 A‐to‐G sites (Figure S1B,C and Table S1). Sample clustering based on RNA editing patterns accurately recapitulated tissue types (Figure 2a), validating the biological relevance of our dataset. The number of RNA editing sites ranged from 2320 in the blastocyst to 96 812 in the brain (Figure 2a), with site counts showing no significant correlations with sample size or mean mapping reads (*p* > 0.05; Figure S2B–D). Moreover, the overall editing activities exhibited a highly edited pattern in blastomere (0.13 ± 0.04) and blastocyst (0.21 ± 0.07), but markedly lower in muscle (0.07 ± 0.02) and the morula (0.04 ± 0.03) (Figure 2b), and these mean editing levels were also not associated with mean sequencing depth across tissues (*p* > 0.05; Figure S2D). At the global level, samples exhibited clear tissue‐dependent separation based on RNA editing profiles (Figure 2c), in line with tissue‐specific editing patterns previously reported in human GTEx [26, 27].

Furthermore, we observed that RNA editing enzymes displayed distinct and tissue‐specific expression patterns across pig tissues, with ADARs and APOBECs showing markedly different distributions (Figure S2E). To quantify their regulatory contribution, we assessed the correlation between enzyme expression and overall editing levels. *ADARB1* explained up to 40% of the editing variation in the kidney and hypothalamus, while *APOBEC2* accounted for 39% in the heart and frontal cortex (Figure 2d; Figure S2F). While RNA editing enzymes could partially explain the occurrence of RNA editing, the differing clustering patterns observed between RNA editing and the enzymes in tissue correlations indicated that RNA editing was subject to regulation beyond the influence of RNA editing enzymes (Figure S2G,H).

### 2.2 Characterization of Edited Sites

To characterize the RNA editome, we investigated the distribution of RNA editing across the genome. A significant majority of RNA editing events were found within the intronic regions (52.9%) and within the 3'‐untranslated regions (3' UTRs; 19.12%), while a only 2.62% of the edits were detected within the exonic sequences (Figure S3A). Clear disparities in the genomic distribution of sites existed across different tissues. While introns remained the predominant location, 3'‐untranslated regions (3'UTRs) exhibited strong tissue‐specific enrichment, accounting for more than 50% of RNA editing sites in the duodenum (∼2.6‐fold enrichment), cartilage (∼3.0‐fold), and synovial membrane (∼2.7‐fold). The proportion of sites in the exonic areas was relatively smaller but high enrichment (approximately 7% to 16%) in several tissues, including blastocyst (∼6.1‐fold), embryo (∼4.9‐fold), cartilage (∼4.1‐fold), and ileum (∼4.1‐fold) (Figure 2e; Table S2). Furthermore, the variants in exonic regions predominantly lead to non‐synonymous amino acid changes (57.6%) (Figure 2f; Figure S3B).

Additionally, a substantial portion of the editing, amounting to 217 676 sites, took place within the porcine repetitive element (PRE) elements, of which approximately 74% of the sites were located in the Pre0_SS element, highlighting a genomic area of concentrated RNA editing activity in pigs (Figure 2g), which were consistent with previous studies [28, 29]. Our further investigation into the editing patterns across 34 different tissues revealed that RNA editing events in pigs exhibited tissue specificity. We specifically sought out tissue‐specific RNA editing sites and discovered 2473 sites that were subject to exclusively or preferentially editing within a single tissue type, with embryo, pituitary, and blastocyst showing the highest overall RNA editing levels (Figure 2h; Figure S3C and Table S3). Further co‐editing network analysis revealed 10 modules that exhibit clusters of similar RNA editing sites level patterns across distinct tissues (Figure 2i; Figure S3D). Among these, 115, 110, 260, 249, 107, 84, and 42 hub sites were respectively observed in the yellow‐oocyte, green‐synovial membrane, blue‐milk, brown‐artery, red‐fetal thymus, black‐spleen, and pink‐frontal cortex modules, with a high correlation between each module and its corresponding tissue (*r* >0.8, *p* < 0.05) (Table S4). Functional annotation of these tissue‐specific sites and modules revealed a concordance with physiological roles — such as PPAR signaling in the adipose and synaptic signaling in the frontal cortex — indicating that tissue‐specific RNA editing aligns with and potentially influences tissue‐specific biological programs (Figure S4).

### 2.3 Identifying edQTL Across the Pig Genome

To uncover the potential genetic variations that can explain RNA editing, we conducted edQTL analysis using 3087 268 common genomic variants from the PigGTEx project [16]. To identify and control for potential confounding factors of editing‐level measurements, we performed principal component (PC) analysis. The significant PCs, determined by a Bayesian empirical approach with a significance level of *p* < 0.05, collectively contributed an average of 41.4% of the total variance in RNA editing levels. Moreover, the top PCs captured the majority of breed‐associated variance and expression level of *ADAR* (Figure S5A–C). Subsequently, we identified edQTLs within the pipeline [16] by considering PCs described above in each tissue.

**FIGURE 3:**
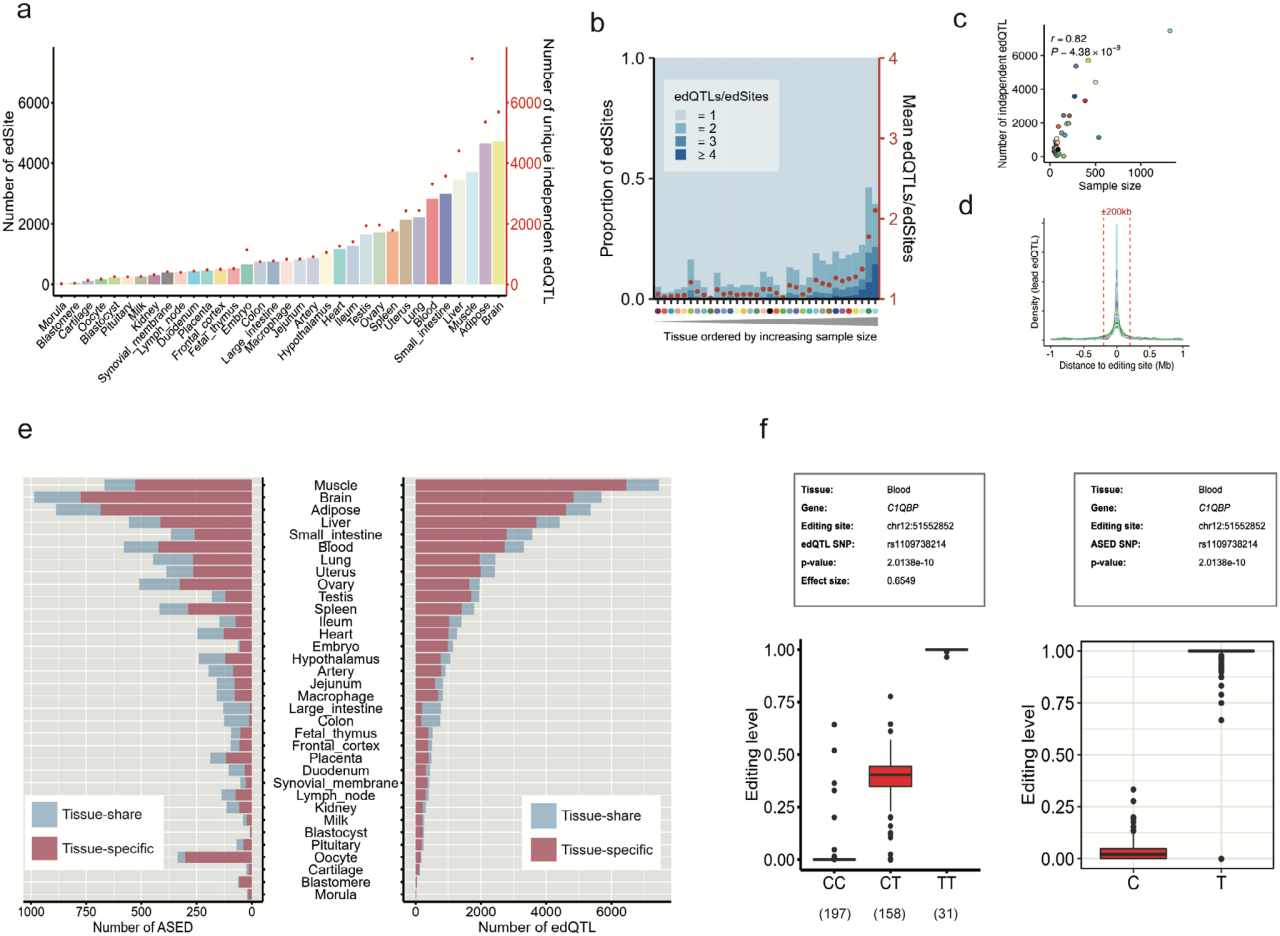
Identification of *cis*‐regulated RNA editing by edQTL and ASED analysis in multiple tissues. (a) number of edSites and unique independent edQTLs in each tissue. (b) Distribution and the average number of independent edQTL per editing site. (c) Pearson's *r* between the number of independent edQTLs and sample size across 34 tissues. (d) The density of independent edQTLs within ±1 Mb of their respective edSites. Vertical dotted lines indicate ± 200 kb relative to the editing site. (e) Histograms of the number of ASED (left) and edQTLs (right) in multiple tissues. (f) Example of an edQTL site in the *C1QBP* gene.

We identified 20 463 (6.48%) unique RNA editing sites (edSites) harboring 49 614 independent cis‐edQTLs (range: 18–7453 per tissue), among which ∼11.6% were associated with multiple independent edQTLs across tissues (Figure 3a,b; Table S5). The number of edSites and independent edQTLs was positively correlated (Pearson's *r* > 0.6, *p* < 1×10^−4^) with sample size across tissues (Figure 3c; Figure S5D), consistent with the trends observed in other molecular phenotypes^19^. Moreover, we observed that the lead edQTLs (permutation‐based false discovery rate of < 0.05, see Methods) were mainly clustered around RNA editing sites, showing increased significance with closer proximity to these sites (Figure 3d; Figure S5E). Additionally, the lead SNPs of edSites were significantly closer to the RNA editing sites and had a greater effect compared to those of non‐edSites (Figure S5F).

### 2.4 Analyzing Allele‐Specific RNA Editing (ASED)

To verify edQTLs, we also conducted an (allele‐specific RNA editing) ASED analysis. We identified 4139 unique allele‐specific RNA editing sites corresponding to 4481 unique SNP (Table S6). To further explore the tissue specificity and sharing of genetic regulation, we assessed the tissue distribution of edQTLs and ASED signals. We observed 33 270 (91.14%) edQTLs and 4030 (89.94%) ASED SNP sites were present in only one tissue (tissue‐specific), and 3234 (8.86%) edQTL and 451 (10.06%) ASED sites were observed in two or more tissues (tissue‐share) (Figure 3e; Figure S5G). As an example of tissue‐specific regulation, we identified an A‐to‐G RNA editing site that was strongly associated with a genetic polymorphism was seen in the 3’UTR of the *C1QBP* gene, where the C‐allele had an average 0.04 RNA editing level and the T‐allele had an average 0.96 RNA editing level, which were comparable to the values for the homozygous CC (0.01) and TT (0.99) genotypes in the edQTL analysis (Figure 3f). Another example of tissue‐specific regulation was an A‐to‐G RNA editing site in intergenic between *SHH* and *CNPY1* associating with a genetic polymorphism in six tissues (brain, adipose, blood, liver, lung and testis), where A‐allele had an average RNA editing level of 0.21, and G‐allele had an average RNA editing level of 0.67, and this result was consistent with edQTL (Figure S5H).

### 2.5 Functional Characterization of edQTL in Pigs

**FIGURE 4:**
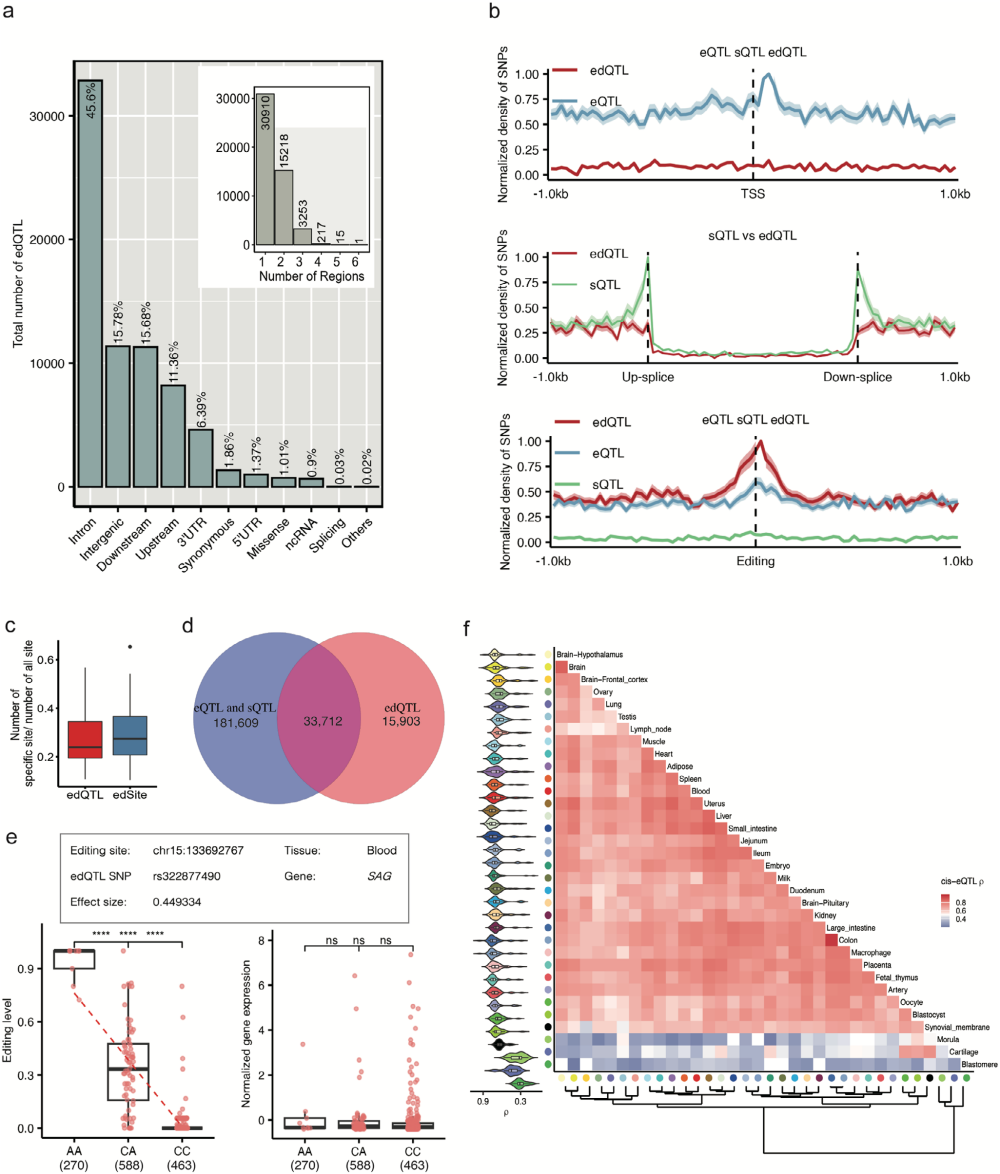
Characterization of edQTLs in the Pig. (a) The proportion of edQTLs is broken down by genic region. The inset plot counts the number of edQTLs commonly detected across more than one region. (b) Comparing the distribution of SNPs acting as eQTLs, sQTLs, and edQTLs around the regions of gene bodies, splice junctions, and edSites. (c) The box plot of number of type‐specific sites (not including in sQTLs and eQTLs but including in edQTLs) divided by all sites across tissues. (d) The venn of type‐specific edQTLs that exist in edQTLs but not in eQTLs and sQTLs. (e) Box plot of the significant association of rs322877490 with the editing level at chr15: 133692767 editing levels and the *SAG* gene expression levels within the blood. (f) effect strengths of edQTL shared in pairs across tissues, with color shades reflecting the spearman correlation (*ρ*) between the effects of the paired edQTL. The violin plot on the left indicates the distribution of spearman's *ρ* between the target tissue and other tissues.

To annotate the edQTL, we investigated the distribution characteristics of these edQTLs across the genome. We found that 45.6% of edQTLs were enriched within intronic regions, followed by intergenic regions, and the proportion of exonic edSites regulated by edQTLs increased in several tissues compared to all RNA editing sites, reaching as high as 26.9% in the morula (Figure 4a; Figure S6A). This suggests that RNA editing in exonic regions may play a more prominent role in early embryonic development. Next, we compared edQTLs with eQTLs and sQTLs identified in the same dataset. Consistent with their regulatory roles, eQTLs showed a strong enrichment around the transcription start sites (TSSs) of protein‐coding genes, sQTLs clustered around splice junctions, whereas edQTLs exhibited a pronounced enrichment in the regions immediately surrounding RNA editing sites (Figure 4b). In terms of regulatory magnitude, edQTLs displayed significantly larger effect sizes than eQTLs (median absolute slope: 0.54 vs. 0.36; Wilcoxon test *p* < 2.2 × 10^−^^1^ ^6^), though smaller than sQTLs (Figure S6B). These distinct spatial patterns highlight different regulatory architectures for eQTLs, sQTLs, and edQTLs.

In addition, we found 15 903 (32.05%) unique edQTLs of 10 035 (49.04%) unique edSites were not found in sQTL and eQTL after considering linkage disequilibrium (LD) among SNPs (*R^2^* <0.8) (defined as type‐specific edQTLs) (Figure 4c,d; Table S7). The case of *SAG* in the blood showed that while the editing level at chr15:133692767 differed significantly among genotypes (*p* < 0.0001), gene expression remains unchanged across genotypes (Figure 4e). Further investigation into the functional impact of edQTLs revealed that the effect sizes of type‐specific edQTLs were generally smaller than those of non‐type‐specific edQTLs across most tissues (Figure S6C). These findings suggest that edQTLs represent a distinct regulatory layer that cannot be fully captured by eQTL and sQTL analyses alone. A total of 498 edQTL‐editing pairs, each involving type‐specific edQTL, were identified across more than two tissues (Figure S6D).

To further investigate the extent of tissue sharing, we conducted a meta‐analysis and found that *cis*‐genetic effects on edSites are largely shared across tissues, with effect correlations exceeding 0.75 in 26 tissue pairs, except in the morula and the blastomere, where patterns diverged (Figure 4f). We also examined tissue‐specific edQTLs using 2473 previously identified tissue‐specific RNA editing sites (Figure 2h) and found that 949 edQTL‐edSite pairs were in a single tissue (Figure S6E and Table S8). These results demonstrate that while edQTLs are largely shared across tissues, the presence of tissue‐specific effects and their partial uncoupling from tissue‐specific editing reflects the complexity of RNA editing regulation.

### 2.6 edQTLs Influence Complex Traits

**FIGURE 5:**
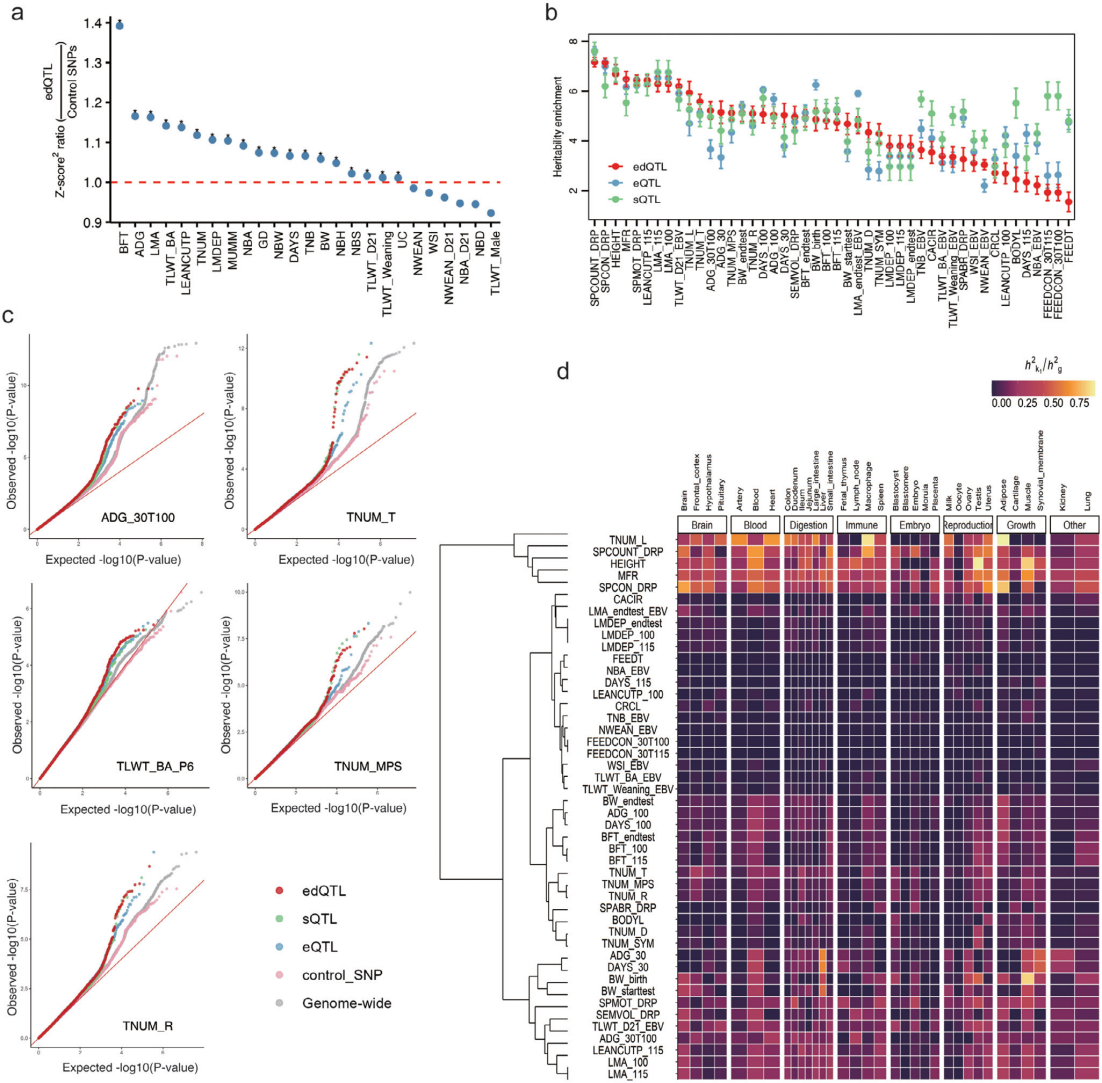
The involvement of edQTLs in the complexity of traits. (a) Enrichment of edQTLs in GWAS signals across 25 complex traits. Point represents the ratio of mean‐squared Z‐scores for edQTLs versus matched control SNPs. A Ratio >1 indicates a stronger association. Red asterisks denote traits with significant enrichment (empirical *p* < 0.05). Based on 1000 permutations, ^*^p < 0.05. (b) Enrichment of heritability for 46 traits mediated by edQTL, eQTL, and sQTL, with values transformed by the log of enrichment. (c) Quantile‐Quantile plots of *P* Values for GWAS SNPs marked as edQTLs, sQTLs, and eQTLs. (d) Percentage of heritability estimates mediated by edQTL across 34 tissues.

Aiming to evaluate the significance of edQTL enrichment in complex trait associations, we performed permutation‐based enrichment tests across 25 traits. In 76% (19/25) of the tested traits, edQTLs exhibited significantly higher GWAS Z‐scores than matched control SNPs (empirical *p* < 0.05, Figure 5a; Figure S7A), indicating a non‐random and functionally meaningful role for RNA editing in phenotypic variation.

We further quantified the cumulative genetic contribution of edQTLs to these complex traits using heritability enrichment analysis. Like eQTLs and sQTLs, edQTLs can also account for part of the heritability of complex traits. Among the 46 traits with sufficient GWAS power (sample size >1000), significant enrichment of edQTL‐associated heritability was observed in 71.7% (33/46) of traits (enrichment > 1‐fold, *P* <0.05), with edQTL enrichment exceeding that of both eQTLs and sQTLs in 39% (18/46) of traits (Figure 5b; Table S9). These findings indicate that edQTLs capture a distinct and frequently predominant component of the genetic architecture underlying complex traits. Consistently, QQ plots revealed a stronger inflation of GWAS signals for edQTLs compared to matched control SNPs across several traits such as average daily gain (30–100 kg) (ADG_30T100), platelet distribution width (PLDWID), litter weight, piglets born alive (TLWT_BA_P6), teat number, maximum per side (TNUM_MPS) and right teat number (TNUM_R) (Figure 5c; Figure S7B), further supporting the enrichment of trait‐associated variants among edQTLs.

To dissect tissue‐specific regulatory contributions, we examined trait‐associated enrichment patterns across tissues and identified 35 significant tissue–trait pairs involving 14 tissues and 23 traits, where edQTLs were enriched while neither eQTLs nor sQTLs showed significant association (Figure S8). For example, 981 edQTLs showed enrichment in the ADG_30T100 trait in the lymph node, while sQTLs and eQTLs did not show enrichment for this trait in the same tissue (Table S9). Aggregating contributions across tissues, edQTLs substantially contributed to the heritability of reproductive and growth traits, including left teat number (TNUM_L), sperm count (SPCONT_DRP), body height (HEIGHT), meat to fat ratio (MFR), and sperm concentration (SPCON_DRP) (Figure 5d). Taken together, these findings demonstrate that edQTLs contribute significantly to the genetic architecture of complex traits in pigs.

### 2.7 Revealing Shared Genetic Regulation Between RNA Editing and Complex Traits

To determine whether RNA editing and complex traits are influenced by shared genetic variants, we performed colocalization analysis using summary statistics from 268 meta‐GWAS studies spanning 232 traits. Applying stringent criteria (posterior probability > 0.9), we identified widespread colocalization between edQTLs and GWAS signals across multiple populations. In total, we detected 3785 colocalization events between edQTLs and GWAS signals in at least one tissue, involving 1000 unique edSites and 120 complex traits (Figure S9A; Table S10), supporting a shared genetic basis between RNA editing and phenotypic variation.

**FIGURE 6:**
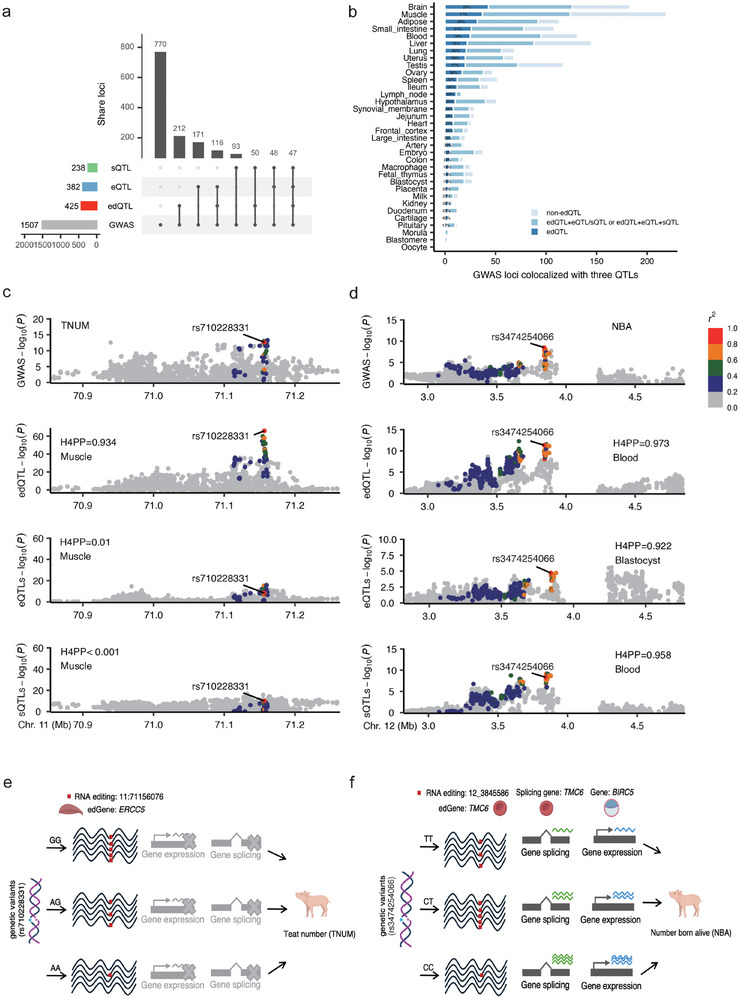
Colocalization analysis between edQTL, eQTL, or sQTL and GWAS signals. (a) Upset plot for GWAS loci explained by different QTL in the cross‐species uniform trait classification trait. (b) Stacked bar chart of GWAS loci colocalized with non‐edQTLs, edQTLs combined with eQTLs and/or sQTLs, and exclusive edQTLs across different tissues. The tissues are ranked by the percentage of edQTLs among all QTLs. (c) Example of an edQTL event in *ERCC5* with colocalizing edQTL signals and non‐colocalizing eQTL and sQTL signals in the muscle for TNUM trait. H4PP, H4 posterior probability. (d) Example of an edQTL event in *TMC6* with colocalizing edQTL and sQTL signals in the blood and colocalizing eQTL signals in the blastocyst for NBA trait. (e) At the rs710228331 locus, the SNP affects the RNA editing level of *ERCC5* in muscle and may further influence TNUM. (f) At the rs3474254066 locus, the SNP alters the RNA editing of *TMC6* in blood and also affects *BIRC5* expression or splicing in the blastocyst, and may further affect to NBA.

To determine whether edQTLs regulate traits independently or in conjunction with other QTLs, we analyzed the colocalization of edQTLs, eQTLs, and sQTLs with GWAS signals at the same GWAS loci (Figure S9B–D). Among the 425 loci (28.2% of tested GWAS loci) where edQTLs colocalized with GWAS signals, 213 (50.1%) also colocalized with either eQTLs or sQTLs (Figure 6a; Table S11). Among these cases, we found that edQTL and eQTL/sQTL signals converged on the same target gene in only 59 cases (32 sharing the same lead variant) and different genes in 154 cases, indicating largely distinct regulatory effects (Figure S9E,F; Table S12).

To further dissect the regulatory contributions of edQTLs across tissues, we examined their colocalization with GWAS loci in 34 tissues. Tissues such as lymph node (69%) and cartilage (62%) showed high proportions of edQTL‐only colocalizations (Figure 6b), suggesting a prominent role for RNA editing in trait regulation in these tissues. In multiple tissues, a considerable proportion of GWAS‐associated loci involving edQTLs also showed colocalization with eQTLs or sQTLs, including 63.2% in embryo, 58.8% in hypothalamus, and 53.4% in blood, suggesting coordinated regulatory effects across different molecular layers (Figure S9G). Consistent with these patterns, edited genes by tissue‐specific edQTL–GWAS colocalizations showed well‐established functions in biological processes relevant to the corresponding tissues (Table S13).

We specifically highlighted a significant edQTL (rs710228331) that strongly influences RNA editing at exonic position chr11:71156076 of the *ERCC5* gene in the muscle (*p* = 1.19 × 10^−^^66^) (Figure S10A). This variant showed high colocalization with a GWAS signal associated with teat number (TNUM) (posterior probability for shared causal variant, H4PP = 0.934), and the strongest shared signal was observed between the GWAS and edQTL, rather than with the corresponding eQTL or sQTL (Figure 6c). *ERCC5* encodes a nucleotide excision repair enzyme involved in DNA damage repair, cell cycle control, and development [30], and muscle could indirectly regulate mammary development or teat formation through secreted hormones or myokines [31]. These findings suggest that RNA editing of ERCC5 in muscle may play a role in the genetic regulation of TNUM (Figure 6e).

Additionally, for the reproductive trait of number born alive (NBA), we identified another locus (rs3474254066) where edQTL, eQTL, and sQTL each showed significant colocalization with the GWAS signal (Figure 6d). This variant affected RNA editing at chr12:3845586 downstream of the *TMC6* gene and influenced alternative splicing in the blood, as well as the expression of *BIRC5* in the blastocyst (Figure S10B). *TMC6* is a protein‐coding gene involved in innate immune responses [32]. *BIRC5* plays a key role in porcine reproductive development, and Jeong et al. reported that supplementing porcine oocyte maturation with allicin increased *BIRC5* expression and improved oocyte developmental potential [33]. These multi‐layered regulatory associations suggest that the locus may influence NBA through coordinated modulation of RNA editing, splicing, and gene expression (Figure 6f).

Taken together, our findings underscore the complementary value of edQTL analysis in elucidating the complex mechanisms of genetic regulation beyond traditional eQTL or sQTL frameworks.

### 2.8 Comparison of RNA Editing Between Pigs and Humans

**FIGURE 7:**
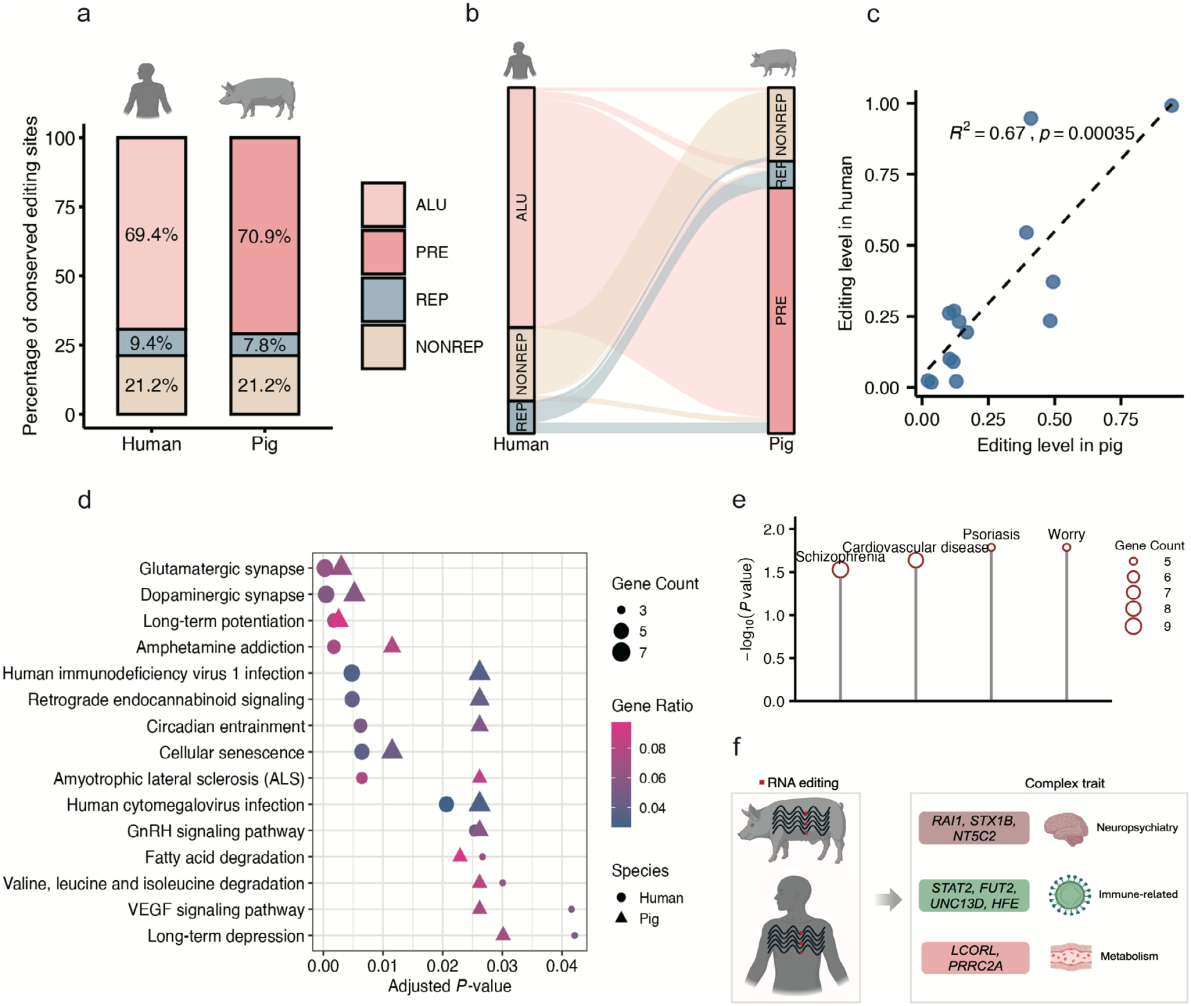
Conservation of RNA editing and genes between the pig and human. (a) Proportional distribution of conserved RNA editing sites in humans and pigs across different genomic contexts: ALU and PRE, other repeat elements (REP), and Non‐repetitive regions (NONREP). (b) Sankey diagram showing the correspondence of conserved RNA editing sites between human and pig repeat categories. (c) Pearson correlation of RNA editing levels for conserved sites in the brain between pigs and humans. (d) KEGG pathway enrichment of genes harboring conserved RNA editing sites in humans (circles) and pigs (triangles). Color indicates gene ratio; size reflects gene count. (e) Enrichment of GWAS traits in human orthologs of pig conserved editing‐host genes that are associated with human edQTL‐GWAS Loci (*P* <0.05). (f) Representative homologous genes underlying the GWAS trait enrichments shown in (e). These genes are involved in conserved RNA editing in pigs and associated with complex trait loci via edQTL in humans.

To investigate the conservation of RNA editing, we compared the RNA editing sites between pigs and humans using the LiftOver tool and sequence conservation scores based on phastCons [34, 35, 36]. Among 14 113 pig RNA editing sites mapped to the human genome, 320 sites (2.27%, 4.46‐fold enrichment, empirical *p* < 0.001) were also detected as editing events in humans (Table S14). Among these conserved sites, approximately 21.2% of sites were located in non‐repetitive regions in both species, while the remaining sites predominantly resided within species‐specific SINE elements—ALU in humans (69.4%) and PRE in pigs (70.9%) (Figure 7a). Further cross‐species mapping of conserved editing events showed editing sites embedded in human ALU elements frequently corresponded to pig PRE regions (Figure 7b), indicating that distinct repeat families may serve analogous roles in maintaining editing activity across species, which was consistent with previous studies. Notably, for these conserved sites, we observed a strong positive correlation in editing levels between pig and human brain tissues (*R^2^* = 0.67, Figure 7c), suggesting that the regulation of these events is preserved.

To investigate potential biological roles of conserved editing‐regulated genes, we performed KEGG pathway enrichment analysis on editing‐host genes conserved between pigs and humans. The analysis revealed significant enrichment in pathways associated with neuronal signaling and plasticity, including glutamatergic synapse, dopaminergic synapse, and long‐term potentiation. These pathways are known to be tightly regulated at the post‐transcriptional level, consistent with potential roles for conserved editing events in modulating neural transcriptomes [37]. Additional enrichment in pathways such as VEGF signaling and fatty acid degradation suggests involvement in metabolic and vascular functions (Figure 7d).

To evaluate the potential phenotypic relevance of editing regulation, we tested whether human orthologs of genes under conserved editing regulation were enriched in known edQTL‐GWAS overlaps [24]. We identified four significantly enriched GWAS traits (worry, psoriasis, cardiovascular disease, and schizophrenia) based on hypergeometric testing (Figure 7e; Table S15). These traits span neuropsychiatric, immune‐related, and cardiometabolic domains, implying that evolutionarily conserved RNA editing regulation may influence a diverse range of human physiological processes. Representative conserved editing‐regulated genes associated with these traits included *NT5C2*, *STAT2*, *FUT2*, and *PRRC2A*, highlighting candidate genes through which editing may contribute to trait variation across species (Figure 7f).

## 3 Discussion

RNA editing serves as a crucial post‐transcriptional mechanism that expands transcriptomic and proteomic diversity. While its significance in human biology is well documented, especially within Alu elements [29, 38, 39], knowledge of RNA editing in pigs has remained limited. In this study, we provide the first comprehensive atlas of RNA editing and its genetic regulation across 34 porcine tissues, thereby filling a key gap in functional genomic resources for livestock species.

Our systematic identification of RNA editing sites revealed widespread and tissue‐specific editing activity in pigs, consistent with patterns observed in other mammals [26, 27]. Importantly, we established a dedicated computational workflow optimized for detecting editing events in pigs using RNA‐seq data alone, thereby providing a practical framework for RNA editing studies in non‐model organisms. By integrating genotype data, we mapped edQTLs that regulate editing levels in a tissue‐dependent manner. Unlike eQTLs and sQTLs, edQTLs cluster proximally to RNA editing sites, suggesting they modulate editing efficiency by altering local RNA secondary structures or ADAR‐binding motifs rather than promoter activity [22]. Future structural analyses (e.g., using RNAfold) are needed to pinpoint these mechanisms. Moreover, tissue‐specific edQTLs were most abundant in testis, consistent with earlier PigGTEx findings where testis also stood out in analyses of other molecular QTLs [16], implying a key role of molecular regulation in testis‐related traits.

A key finding of this study is the substantial contribution of edQTLs to complex agronomic traits. We demonstrate that edQTLs also explain a larger fraction of trait heritability in some traits—similar to observations made in human studies [22]. Previous studies have found evidence of edQTL co‐regulating complex traits with eQTLs through colocalization studies [24, 25, 40], and we extend this by providing additional evidence for a co‐regulatory role with sQTL. We further nominate rs710228331 for TNUM and rs3474254066 for NBA as two representative mechanistic loci, for which targeted functional validation using CRISPR‐based or isoform‐specific assays will be important to definitively establish the causal role of RNA editing regulation in shaping trait variation. Taken together, our results support an important role for edQTLs in regulating complex traits in pigs.

We identified a low proportion of conserved RNA editing sites between pigs and humans, which is biologically expected in mammals [41]. The pattern arises because the primary substrates for ADAR enzymes—SINE retrotransposons—are lineage‐specific (Alu in humans vs. PRE in pigs) [42]. However, the subset of sites that are conserved appears to be functionally critical. This is supported by the strong correlation of editing levels observed between pig and human brain tissues, which implies that the precise regulation of these specific events might be maintained by purifying selection across species. Moreover, given that certain genes (e.g., *C1QBP*) and RNA editing play an established role in immune regulation across species [42, 43, 44], and that Li et al. [22]. demonstrated edQTL‐mediated modulation of immune responses, our results further emphasize the value of cross‐species comparative studies.

While our findings establish the significance of RNA editing in porcine phenotypic variation, several limitations remain. Technically, the absence of matched DNA sequencing limits the definitive exclusion of rare genomic variants, and variable sample sizes across tissues may constrain statistical power. Although our rigorous filtering revealed robust biological patterns, the inherent heterogeneity of public datasets implies that sequencing depth variations could still influence site detection, warranting future study with more uniform data. Additionally, while our inclusion of non‐canonical editing types enables comprehensive discovery, it may introduce technical artifacts. We advise caution in interpreting these specific events and recommend future verification using higher‐quality datasets. Moreover, since the identified edQTLs are based on statistical inference, experimental validation is essential to establish causality. Finally, limitations regarding the heterogeneity of public data, potential artifacts, and the statistical nature of edQTLs highlight the need for future experimental validation and unified cross‐species analytical frameworks to further refine these findings.

In summary, this study provides a foundational resource for porcine RNA editing and its genetic regulation, offering new perspectives into post‐transcriptional control of complex traits. The comprehensive map of RNA editing sites and edQTLs we present offers valuable references for editing‐related research, particularly within the context of the FarmGTEx framework [13], which aims to translate such foundational discoveries across agricultural species. Further integration with proteomic and functional genomic datasets, along with experimental validation, will be key to fully elucidating the roles of RNA editing in mammalian biology.

## 4 Methods

### 4.1 PigGTEx RNA‐seq Data

A total of 5457 RNA sequencing (RNA‐seq) samples derived from the pig genotype‐tissue expression (PigGTEx) [16] project constitute the core data input for the RNA editing detection pipeline we have established. All RNA‐sequencing data were processed and mapped to the Sscrofa11.1 (v100) pig reference genome using star (v2.7.0) [45], and PCR duplicate reads were further removed from the data using the picard tools for the next detection [46].

### 4.2 Detection of Mismatches and Filtering for RNA Editing in Pigs

De novo editing detection by REDItools is stringent [47]. Here, we used REDItools (v1.2.1) to preliminarily identify variants at the RNA level [48, 49]. Moreover, we conducted several filtering steps to retain only high‐quality and high‐confidence reliable RNA editing sites [50, 51]: i) Required the reads of high mapping quality and high base quality; ii) Excluded sites in homopolymer runs of ≥ 5 Bp and truncated the first 6 bases of each read; iii) Removed all known SNPs from dbSNP (v100) and pig genomics reference panel (PGRP) [16]; iv) Implemented different and more stringent filters in different region (PRE family region and non‐PRE region) due to the rich characteristics of the RNA editing sites in the PRE region [28, 29, 52]. At the individual‐sample level, the number of detected candidate sites increased with sequencing depth (Figure S2A), and all cross‐tissue analyses were performed on stringently filtered RNA editing sites to minimize sequencing‐depth–related bias. We tested a range of presence thresholds for each tissue, motivated by the fact that true RNA editing loci typically exist in multiple individuals [53]. For each threshold, we recorded the total number of retained mismatches and the proportion of A‐to‐G substitutions. We observed that the fraction of A‐to‐G mismatches plateaued at a high level when a given mismatch occurred in ≥ 20 samples and in ≥ 10% of the samples for that tissue (Figure S1A). Therefore, we retained the final RNA editing sites that present in ≥ 20 samples and ≥ 10% of the samples per tissue. The details of this pipeline are illustrated in Figure S1C as a flowchart.

### 4.3 BLAT‐Based and Annotation‐based Filtering

Many mismatched sites are caused by sequencing reads being incorrectly aligned to highly similar regions in the reference genome [54]. To eliminate false positives caused by this alignment error, studies often employ blat to realign reads carrying mismatches to the reference genome, Thereby filtering out sites mistakenly identified as mismatches due to high similarity in certain genomic regions [55]. However, similarity comparison analysis using the blat tools is time‐consuming and labor‐intensive, making them unsuitable for large‐scale PigGTEx analysis. Here, we adopted a BLAT‐based strategy originally developed for human GTEx RNA editing data [27]. Specifically, we implemented genome‐wide BLAT simulations in pigs to identify highly similar regions within the genome: i) Divided the reference genome (*Sscrofa11.1*) sequence into 76 Bp‐long subsequences with a step size of 19 Bp employing a sliding window approach, and ultimately generated millions of subsequences to simulate sequencing reads; ii) Aligned these subsequences to the reference genome using BLAT (‐stepSize = 5, ‐repMatch = 2253, ‐minScore = 0, ‐minIdentity = 0) [56], and considered subsequences with alignment length ≥61 Bp and ≥94% identity as highly similar regions on the whole genome (Figure S1B). A BLAT‐based genome high similarity reference was produced from this simulation process, which filters mismatches in non‐PRE regions.

Since most of the RNA‐seq data in the PigGTEx project are not strand‐specific and therefore do not accurately assign reads to the sense or antisense strand [22]. Here, we performed strand correction of mismatch loci based on the reference annotation [27, 50, 57]: i) Matched mismatches with the reference genome annotation file used the bedtools tool; ii) Sites on the positive strand maintained their original mutation type, while those on the negative strand were converted to their reverse complement (e.g., T‐to‐C adjusted to A‐to‐G). The reliability of this annotation‐based correction was confirmed by the resulting mutation spectrum, which showed a dominant proportion of canonical A‐to‐G transitions (∼76%) and negligible T‐to‐C artifacts (Figure S1).

### 4.4 Downscaling Cluster Analysis and Annotation for RNA Editing

All RNA editing sites in each tissue were performed Z‐score normalization on the data to ensure consistency. Subsequently, we performed *T*‐SNE dimensionality reduction using the rtsne software package (https://github.com/jkrijthe/Rtsne) to achieve effective clustering of the data, in which the theta parameter was set to 0.25 and other parameters were kept as default. Results were plotted using the ggplot2 package (version 3.2.1). Moreover, all these sites were well annotated by the annovar tool [58]. Furthermore, RNA editing enzymes' expression was downloaded from the PigGTEx release V0 (https://piggtex.farmgtex.org/). Additionally, the editing level was calculated as the ratio of reads supporting the edited base to the total reads covering the site. The editing level for each sample was defined as the ratio of the total number of reads supporting the edited bases to the total number of reads covering all corresponding RNA editing sites in that sample.

### 4.5 Tissue‐specific Editing Analysis and Co‐Editing Network

To identify RNA editing sites specifically edited in specific tissue, we focused on sites present in at least 50 reads in at least 30 samples for each tissue (the remaining 41 279 RNA editing sites). We then applied the entropy R package to calculate the shannon entropy [59] for each RNA editing site across tissues as a measure of tissue specificity. Tissue‐specific RNA editing sites were identified using an outlier detection method, with sites exhibiting an editing level range greater than 10% and a shannon entropy lower than 0.4 considered tissue‐specific [26].

We performed several data preprocessing steps to identify co‐edited sites according to the per‐site editing level averaged across samples with each tissue. First, we removed sites with excessive missing values (≥4 samples, leaving 47 066 sites). Second, we excluded samples with missing values for ≥50% of the sites (18 samples were removed). Third, we filtered out sites with low variability by removing those with a coefficient of variation (CV) < 0.8, resulting in 1939 sites. Finally, we retained sites expressed in at least 10 tissues (30%), yielding 1677 sites. Subsequently, we used the WGCNA R package [60] to estimate the optimal soft threshold for co‐editing network construction, determining a power value of 4 as the best fit, where the scale‐free topology model fit reached *R* ^2^ = 0.88 (Figure S3D). Using this power value and a minimum module size of 30, we identified 10 co‐editing modules.

### 4.6 Functional Enrichment Analysis

To investigate the biological functions of genes containing tissue‐specific RNA editing sites and genes within WGCNA co‐editing modules, we performed kyoto encyclopedia of genes and genomes (KEGG) pathway enrichment analysis. The analysis was conducted using the clusterProfiler R package and KOBAS online software (http://kobas.cbi.pku.edu.cn/). All expressed genes in the dataset were used as the background gene set. Pathways with a *p*‐value < 0.05 were considered significantly enriched. For visualization, the top enriched pathways for each tissue or module were selected and plotted using ggplot2.

### 4.7 Cis‐edQTL Mapping

To capture the full landscape of post‐transcriptional variation and to allow for the potential discovery of non‐canonical regulation, we performed edQTL analysis on all identified RNA editing sites. We identified potential confounding factors in editorial level measurements using pc analysis. First, raw editing‐level measurements were logit‐transformed before regressing out PCs, and then normalized to N (0, 1) distribution across individuals within each tissue. PCs that reached statistical significance (*p* < 0.05) under the bayesian empirical approach were included as covariates in the edQTL mapping, together with PCs estimated following the PigGTEx procedure [16]. The PCs capture potential technical and experimental confounders [16]. To identify the edQTL, we tested SNPs located within 1 Mb of each RNA editing site using tensorQTL (v1.0.3) [61], consistent with the PigGTEx pipeline [16]. False discovery rates (FDR) were estimated from nominal *p*‐values using the benjamini–hochberg procedure. Within each tissue, associations with FDR < 5% were considered significant.

### 4.8 ASED Analysis

To complement the edQTL Analysis, we employed the ASED method developed by park et al. to perform allele‐specific RNA editing analysis on RNA‐seq data [24, 40]. The workflow was conducted as follows: First, RNA‐seq reads were aligned to the reference genome using STAR with the following parameters: –alignEndsType EndToEnd –outSAMattributes NH HI NM MD –outSAMtype BAM Unsorted –outSJfilterOverhangMin 88888 –outFilterType BySJout –outFilterMultimapNmax 20 –outFilterMultimapScoreRange 0 –outFilterMismatchNmax 6 –outFilterIntronMotifs RemoveNoncanonicalUnannotated –alignIntronMax 300000. Subsequently, allele‐specific alignment biases were removed using WASP, and heterozygous SNP alignments were split into two alleles using a python script developed by park et al. [24, 40].

Specifically, the analysis retained only samples with heterozygous genotypes and non‐zero coverage for both alleles. Based on the split alignments, RNA editing levels were calculated separately for each allele. SNPs were associated with RNA editing sites. We adopted a paired replicate statistical framework to detect allele‐specific RNA editing signals at the population level. This framework treats the two alleles as matched pairs and considers multiple individuals sharing specific heterozygous SNPs as replicate samples, while accounting for the estimation uncertainty of RNA editing levels in each individual. The framework models the paired allele‐specific differences in RNA editing levels across replicate samples. Finally, the false discovery rate (FDR) was controlled at 5% using the Benjamini‐Hochberg method, and the RNA editing ratios for each allele were reported.

### 4.9 Characterized edQTLs and Compared with eQTLs and sQTLs

The edQTLs were annotated by snpEff based on ensembl genomic annotation (release 100). Furthermore, we obtained eQTLs and sQTLs mapped by the PigGTE analysis to compare the edQTLs, eQTLs and sQTLs. We procured the TSS for each transcript from the ensembl genomic annotation database. Subsequently, we extended the sequences ±1 Kb surrounding the TSS and partitioned these regions into 40 equal bins to ascertain the density of SNPs for both edQTLs and eQTLs. For each splicing junction, we extended the sequences by ±1 Kb and similarly divided them into 40 equal bins to calculate the SNP density specific to edQTLs and sQTLs. In the case of RNA editing sites, we extended the sequences by ±1 Kb and further divided these into 40 equal bins to determine the SNP density for edQTLs, eQTLs, and sQTLs, respectively. To identify edQTLs that are specific relative to eQTLs and sQTLs, we employed LD analysis. Significant (FDR < 0.05) and independent edQTL, eQTL, and sQTL loci were extracted to generate SNP Lists. PLINK v1.90 was used to analyze genotype data and calculate LD (*R* ^2^) between SNPs. SNP pairs with LD *R* ^2^ ≥ 0.8 were considered linked. EdQTLs with LD *R* ^2^ < 0.8 with eQTLs or sQTLs were retained as type‐specific edQTLs. To exclude the possibility that type‐specific edQTLs were merely shared QTLs with insufficient power for eQTL detection, we retrieved the nominal *P*‐values of these loci from the eQTL summary statistics. The vast majority of these variants (average 87.7% across tissues) exhibited no evidence of association with gene expression (nominal *p* > 0.05), confirming the robustness of our classification strategy.

### 4.10 Tissue‐Shared and Tissue‐Specific edQTLs

To investigate the tissue‐sharing patterns of edQTLs, we integrated two computational tools, MashR (v0.2–6) and METASOFT (v2.0.1) [62, 63], to comprehensively analyze edQTL data across 34 tissues. In the MashR analysis, we constructed a multi‐tissue effect model based on the statistical metrics (slope/slope_se) of the most significant edQTL for each edSite. Through posterior estimation using MashR, we obtained the mean effect size of edQTLs and their significance metric (local false sign rate, LFSR). EdQTLs with LFSR < 0.05 were considered to have significant effects in specific tissues. Additionally, we evaluated the tissue‐sharing patterns of edQTL effects by calculating the rank correlation coefficients of significant edQTL effects between pairwise tissues. In the METASOFT analysis, we input the effect sizes (slope) and standard errors (slope_se) of edQTLs across all tissues and computed the posterior probabilities (M‐values) of edQTLs using a markov chain monte carlo (MCMC) approach. EdQTLs with M‐values > 0.7 were defined as significant, further validating the results from the MashR analysis. To identify tissue‐specific edQTLs, we first defined a set of tissue‐specific RNA editing sites. An editing site was classified as tissue‐specific if it met stringent criteria. This filtering process yielded 2473 tissue‐specific RNA editing sites across all tissues. Subsequently, we retrieved all significant edQTLs (*p* < 0.05) associated with this specific subset of 2473 sites from our primary QTL mapping results. The resulting edQTLs were designated as tissue‐specific edQTLs.

### 4.11 Enrichment Analysis of QTLs in GWAS Signal

To assess whether edQTLs were enriched for *complex* trait associations, we tested if they exhibited stronger GWAS signals than expected by chance for 25 traits with a unified cross‐breed classification. For each trait, we compared the distribution of squared GWAS Z‐scores (Z^2^) between the lead edQTL SNPs and an equally sized set of randomly sampled control SNPs. We then used a one‐sided wilcoxon rank‐sum test to assess if the edQTLs exhibited a significantly higher distribution of GWAS association statistics compared to the control set. Furthermore, we conducted heritability enrichment analysis following the workflow described by Li et al. [22]. First, independent signals were extracted by clustering significant QTLs with PLINK [64]. Using the parameters –clump‐r2 0.4 and –clump‐kb 250. From GWAS signals with QTL annotations in pigs, independent edQTLs, sQTLs, and eQTLs across 34 tissues were extracted, yielding 119 930, 49 259, and 188 255 loci respectively for follow‐up analysis. Second, a negative control site set was constructed using the PGRP V1 genomic reference panel [16, 65].

To assess the enrichment of GWAS signals, we generated sets of matched background SNPs. For each focal edQTL, we selected control SNPs located on the same chromosome that satisfied the following matching criteria: i) With minor allele frequency (MAF) within 0.02 of the focal SNP's MAF, and ii) With linkage disequilibrium (LD) score within 0.1 standard deviation of the focal SNP's LD score. Both MAF and LD scores were calculated using the PGRP reference panel with default parameters [65, 66], which contains 268 meta‐GWAS results from 71 885 pigs, providing a rich source of genetic variation data. GWAS summary statistics data of 46 traits (GWAS sample size >1000) were described in Table S16. Furthermore, based on the experimental data of 6 pig groups (sample size range: 1071–4383), we used the REML (restrictive maximum likelihood) model [67] to calculate the genetic strength of 46 complex traits in the group and evaluated the genetic side of eQTL, sQTL, and edQTL in 34 tissues. We calculated the interpretation ratio of the difference to quantify the contribution of these loci to differences in editing levels. All are built with LDAK software (v5.2) [68]. The following parameters were used in the model construction process: –calc‐kins direct and –power 0 to ensure the accuracy and reliability of the genetic relationship matrix.

### 4.12 Colocalization Analysis

To explore the potential shared causal variants between molecular QTLs and GWAS signals, we performed colocalization analyses using COLOC [69]. With default settings. The analysis included 268 meta‐GWAS datasets covering 207 complex traits across five categories, and molecular QTLs (edQTLs, eQTLs, and sQTLs) were identified across 34 tissues. For each edGene, defined as a gene containing a high‐confidence RNA editing site or residing within the cis regulatory window (±1 Mb) of a significant edQTL–edSite association, we defined a colocalization region as a 1 Mb window centered on the corresponding edSite (±1 Mb flanking the site). This window size was chosen to encompass local linkage disequilibrium (LD) structure, following the convention used in GTEx and PigGTEx. Within each region, we retained GWAS loci only if the minimum *p*‐value of variants within the region was less than 1 × 10^−^^5^, a threshold that balances sensitivity and computational tractability and is consistent with the PigGTEx and GTEx colocalization pipelines. We then identified GWAS loci that overlapped with edQTL signals within these regions. We calculated the posterior probabilities of five hypotheses (H0–H4) and considered H4PP values >0.9 to indicate colocalization of GWAS and edQTL signals. We applied the same method to eQTLs and sQTLs to find shared causal variants between GWAS and molecular QTLs. In this study, we adopted the same GWAS loci selection criteria as PigGTEx [16] by identifying loci centered on lead SNPs (*p* < 1 × 10^−^^5^) among the 3087 268 SNPs that overlapped between GWAS summary statistics and molQTL mappings. A total of 1507 such loci were used for colocalization analysis.

### 4.13 The Conservation Analysis of Pig and Human Editing and Orthologous Genes

To investigate the evolutionary conservation of RNA editing, we mapped pig RNA editing sites to the human genome using a multi‐step pipeline. First, the genomic coordinates of pig RNA editing sites (Sscrofa11.1) were formatted into a BED file. We used the UCSC liftOver tool [70] with the susScr11ToHg38.over.chain file to convert these coordinates to the human reference genome (hg38). To account for evolutionary divergence and improve mapping efficiency, the minimum required sequence match fraction was set to 0.5 (‐minMatch = 0.5). To ensure the identification of high‐confidence conserved sites, we applied two sequential filters to the successfully lifted‐over coordinates. First, we assessed sequence‐level conservation by retaining only sites located in regions with a phastCons 100‐way score greater than 0.5 [71]. Second, to identify sites with functional conservation, these filtered coordinates were intersected with known human RNA editing sites from the REDIportal (v2.0) database [72, 73], and only sites that overlapped between the two species were defined as “conserved RNA editing sites”, which aggregates editing calls across multiple human tissues—rather than strict tissue‐to‐tissue matching to maximize the sensitivity of detecting evolutionary conservation.

To assess the statistical significance of this overlap, we performed a permutation test by randomly sampling 14 113 genomic positions from the human genome 1000 times to determine the expected background overlap (enrichment and empirical *p*‐value calculation). The genomic context of these conserved sites, such as their location within repetitive elements, was analyzed using annotations from RepeatMasker [74]. Orthologous editing‐host genes were identified through ensembl bioMart, and their functional relevance was assessed through KEGG pathway enrichment analysis via the KOBAS online tool [75]. For functional interpretation, we further examined the editing levels of conserved sites in matched brain samples from pigs and humans. In order to assess phenotypic relevance, human GWAS traits associated with edQTL‐linked genes were extracted from published edQTL‐GWAS datasets [24], and enrichment was tested using hypergeometric tests against background gene sets.

## Author Contributions

X. Pan, W. Gong, X. Cai and J. Teng contributed equally to this work. Conceptualization: X.P., Z. Zhang, J.L. and L.F. Methodology and formal analysis: X.P., W.G., X.C., J.T. Investigation and data curation: J.C., Q.S., Z. Zhong, Y.W., W.Z., Y.T. D.X., H.Y., J.H. and T.Z. Software (website construction): H.Z. Supervision: Z. Zhang, J.L., L.F. Writing – original draft: X.P., W.G., X.C., J.T. Writing – review & editing: Z. Zhang, J.L., L.F., Y.G., Y.Z., W.A., X.Y. All authors reviewed, revised, and approved the manuscript.

## Conflicts of Interest

The authors declare no conflicts of interest.

## Supporting information




**Supporting File 1**: advs73841‐sup‐0001‐SuppMat.pdf.

**Supporting File 2**: advs73841‐sup‐0002‐TableS1‐S16.xlsx.

## Data Availability

The data that support the findings of this study are available in the supplementary material of this article.
