# Supplementary material for: Multi‐Tissue Genetic Regulation of RNA Editing in Pigs
Source: Adv Sci (Weinh). 2026 Jan 18;13(17):e18238. doi: 10.1002/advs.202518238 (PMC13042972; doi:10.1002/advs.202518238)
Supplement: Supplementary file 1 — Supporting File 1: advs73841‐sup‐0001‐SuppMat.pdf. [file ADVS-13-e18238-s002.pdf]

SUPPLEMENTAL FIGURES

Multi-tissue Genetic Regulation of RNA Editing in Pigs

Xiangchun Pan[1†], Wentao Gong[1†], Xiaodian Cai[1†], Jinyan Teng[1†], Jiali Cai[1], Haonan Zeng[1], Wondossen Ayalew[1], Qingpeng Shen[1], Zhanming Zhong[1], Yifei Wang[1], Wenjing Zhang[1], Yuhan Tian[1], Dantong Xu[1], The PigGTEx Consortium, Yahui Gao[1], Hongwei Yin[3], Yuebo Zhang[4], Jiahui Hou[1], Tianru Zhou[1], Jiaqi Li[1*], Lingzhao Fang[2*], Xiaolong Yuan[1*], Zhe Zhang[1*]

[1]State Key Laboratory of Swine and Poultry Breeding Industry, Guangdong Laboratory for Lingnan Modern Agriculture, Guangdong Provincial Key Lab of Agro-Animal Genomics and Molecular Breeding, College of Animal Science, South China Agricultural University, 510642 Guangzhou, China
[2]Center for Quantitative Genetics and Genomics (QGG), Aarhus University, Aarhus 8000, Denmark
[3]Shenzhen Branch, Guangdong Laboratory for Lingnan Modern Agriculture, Genome Analysis Laboratory of the Ministry of Agriculture, Agricultural Genomics Institute at Shenzhen, Chinese Academy of Agricultural Sciences, Shenzhen 518124, China
[4]College of Animal Science and Technology, Hunan Agricultural University, Changsha, China

[*]Correspondence: Zhe Zhang (E-mail: zhezhang@scau.edu.cn); Xiaolong Yuan, yxl@scau.edu.cn; Lingzhao Fang (E-mail: lingzhao.fang@qgg.au.dk); Jiaqi Li (E-mail: jqli@scau.edu.cn). Address: College of Animal Science, South China Agricultural University, Guangzhou 510642, Guangdong Province, China.

[†] These authors contributed equally to this article

Supplemental Figures 1-10, in brief:

Fig. S1. The detections of RNA editing based on PigGTEx.

Fig. S2. The multi-tissue RNA editing.

Fig. S3. Characterization of multi-tissue RNA editing.

Fig. S4. Functional enrichment of target genes for RNA editing

Fig. S5. Identification of cis-regulated RNA editing.

Fig. S6. Annotation of edSites and edQTLs in bulk tissue.

Fig. S7. The contribution of edQTLs to the phenotypic variation in complex traits.

Fig. S8. Enrichment of different molecular QTLs in tissue-trait pairs.

Fig. S9. Examining the overlap between edQTL, eQTL or sQTL and GWAS signals.

Fig. S10. Examples of the overlap between edQTL, eQTL or sQTL and GWAS signals

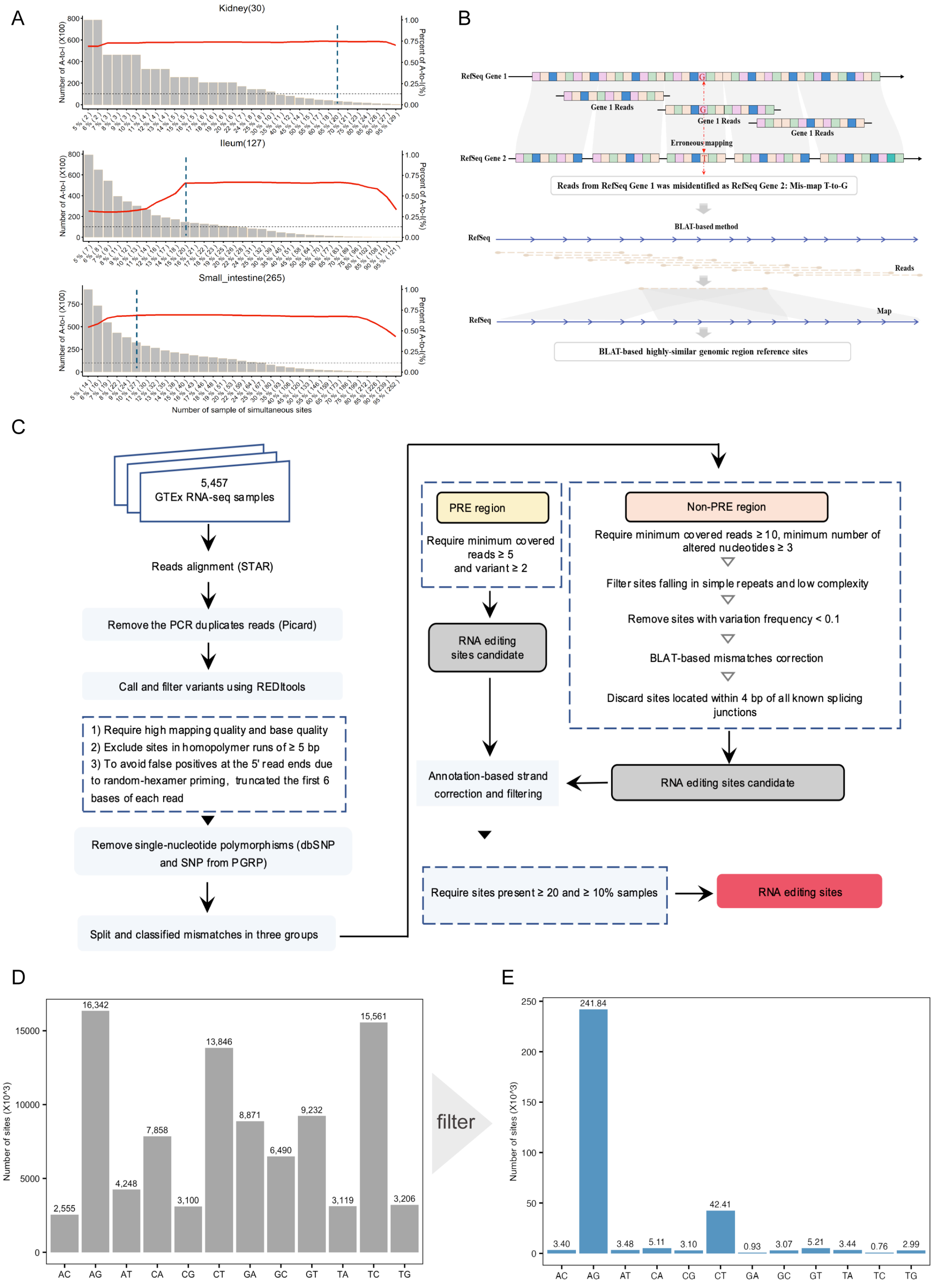

Fig. S1. The detections of RNA editing based on PigGTEx.

(A) Mismatch detection analysis under different sample size gradients. **(B)** Filtering strategies based on BLAT alignment. **(C)** Pipeline for detecting RNA editing in pigs. This process involves multiple filtering steps to retain high-quality and high-confident RNA editing sites. **(D)** Unfiltered distribution of 12 RNA substitution types, indicating the numerous false positive mismatches that necessitate further filtration. **(E)** Distribution of the 12 possible substitution types after stringent filtrations, where ADAR-derived A-to-I (A-to-G) substitutions constitute ~75.28% and APOBEC-derived C-to-U (C-to-T) substitutions ~13.43% of all mismatches.

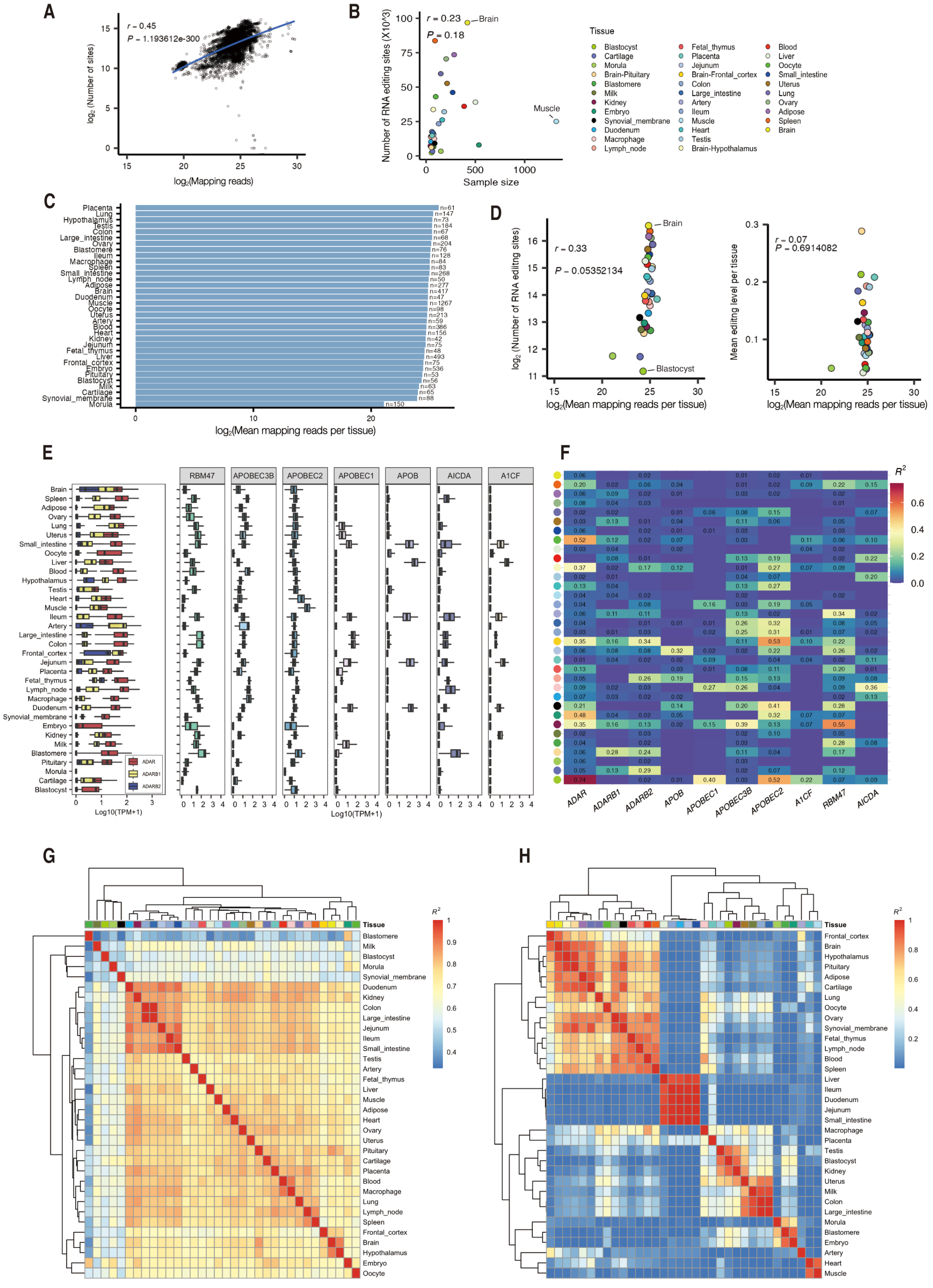

Fig. S2. The multi-tissue RNA editing.

(A) Correlations between the number of RNA editing sites and the mapping reads. **(B)** Pearson correlations between sample sizes and number of RNA editing. **(C)** Average number of mapped reads per tissue. **(D)** Pearson correlation between mean mapping reads per tissue ($\log_2$-transformed)

and RNA editing (editing number or editing level). **(E)** The expressions of *ADAR*, *ADARB1*, *ADARB2, RBM47*, *APOBEC3B, APOBEC2, APOBEC1, APOB, AICDA* and *A1CF*. **(F)** Pearson correlations between expression levels of RNA editing enzymes/relevance-factors and overall editing level in 34 tissues. **(G)** Pearson correlations on the editing levels of 34 tissues, calculated based on the overall editing levels for all sites. **(H)** Pearson correlations on the expression levels of RNA editing enzymes among 34 tissues, calculated using the expression of *ADAR*, *ADARB1*, *ADARB2*, *APOB*, *APOBEC1*, *APOBEC3B*, *APOBEC2*, *A1CF*, *RBM47* and *AICDA*. The color codes for tissues are the same as in **B** throughout.

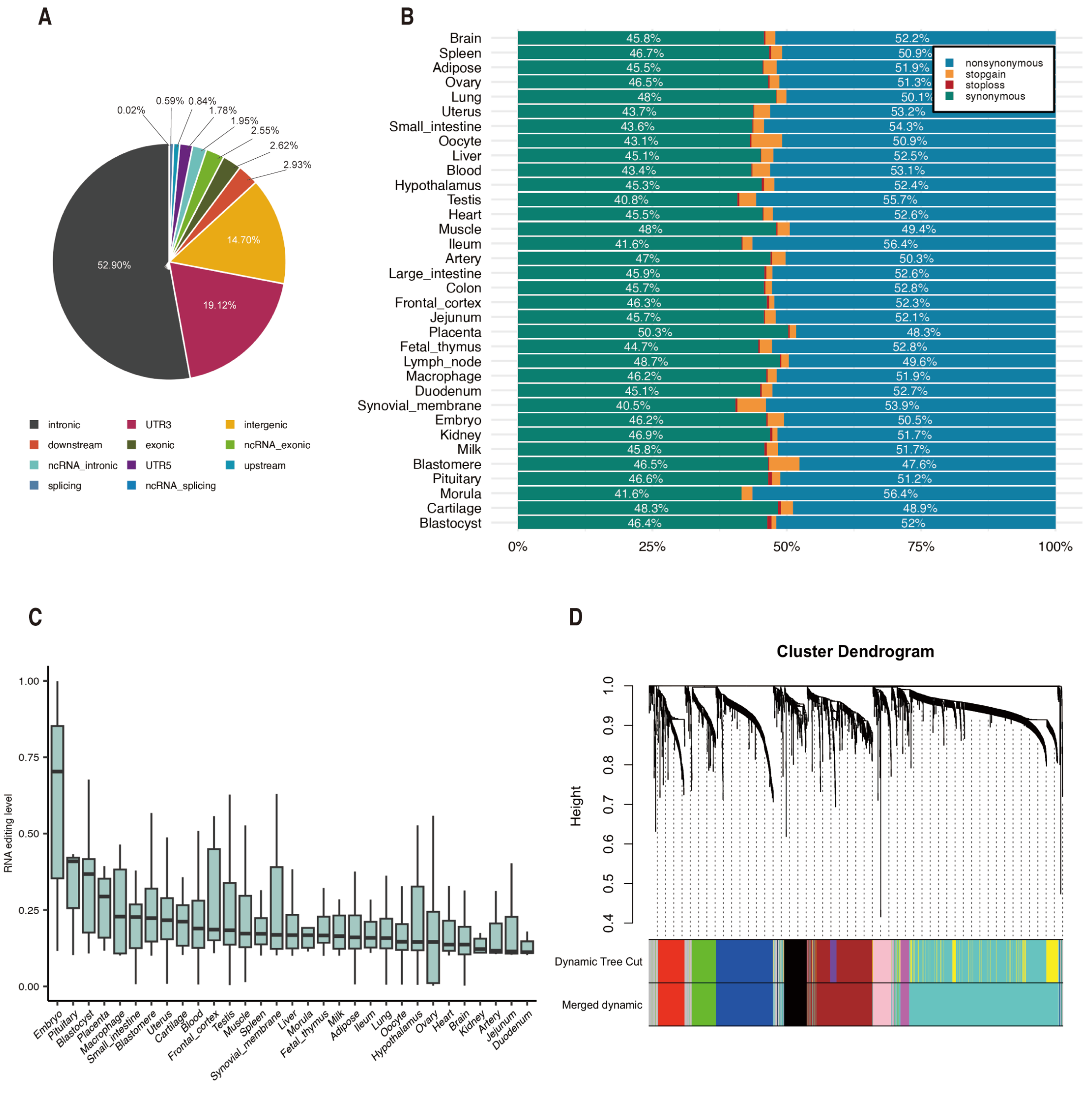

Fig. S3. Characterization of multi-tissue RNA editing.

(A) Proportions of RNA editing in genomic regions. **(B)** Distribution characteristics of exonic variant functions in different tissues, with only the top two proportion displayed in each tissue. **(C)** Editing levels of tissue-specific RNA editing sites across tissues. **(D)** The cluster of modules for co-editing sites.

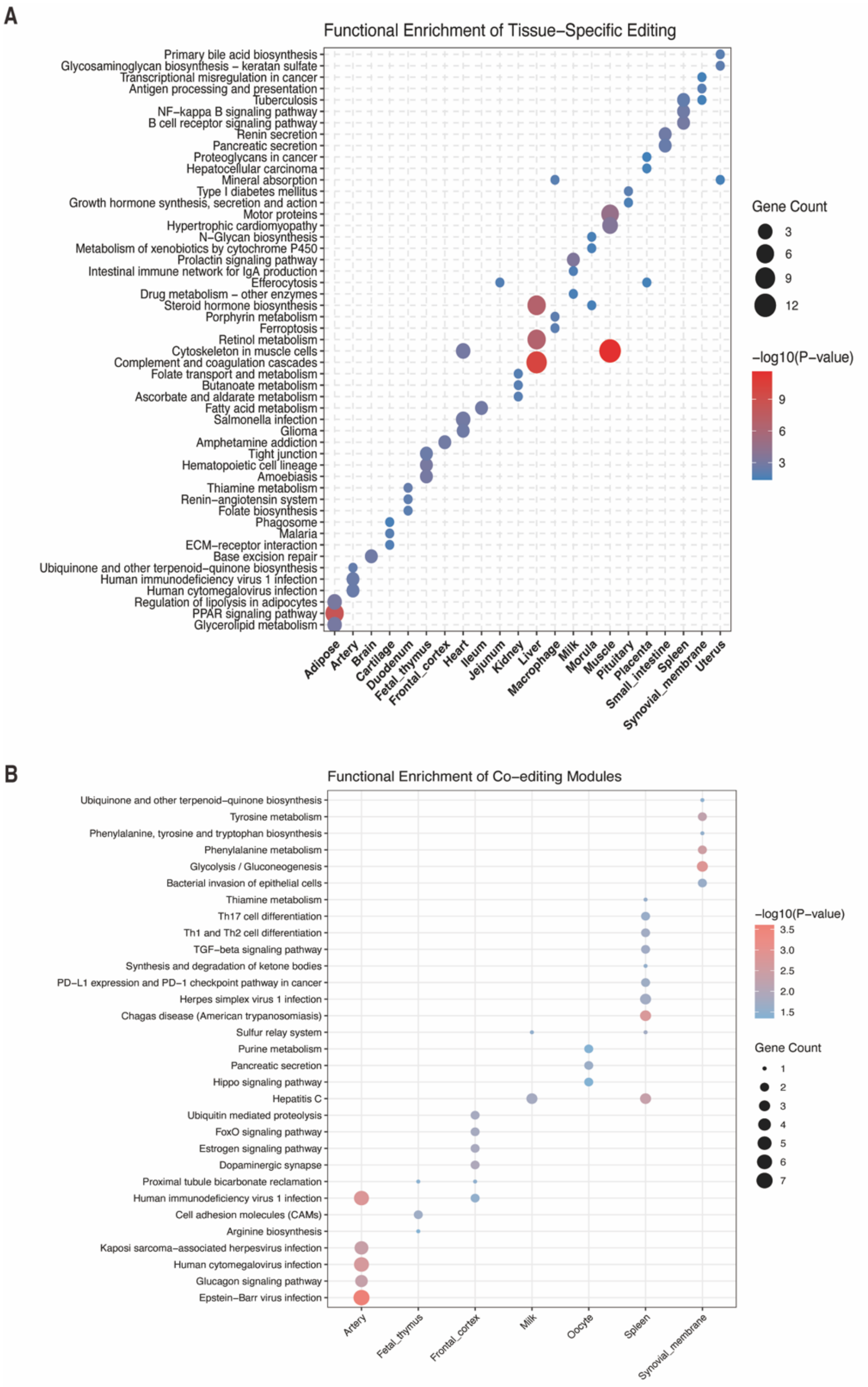

Fig. S4. Functional enrichment of target genes for RNA editing.

(**A**) Functional enrichment of target genes for tissue-specific editing. (**B**) Functional enrichment of target genes for co-editing in tissues.

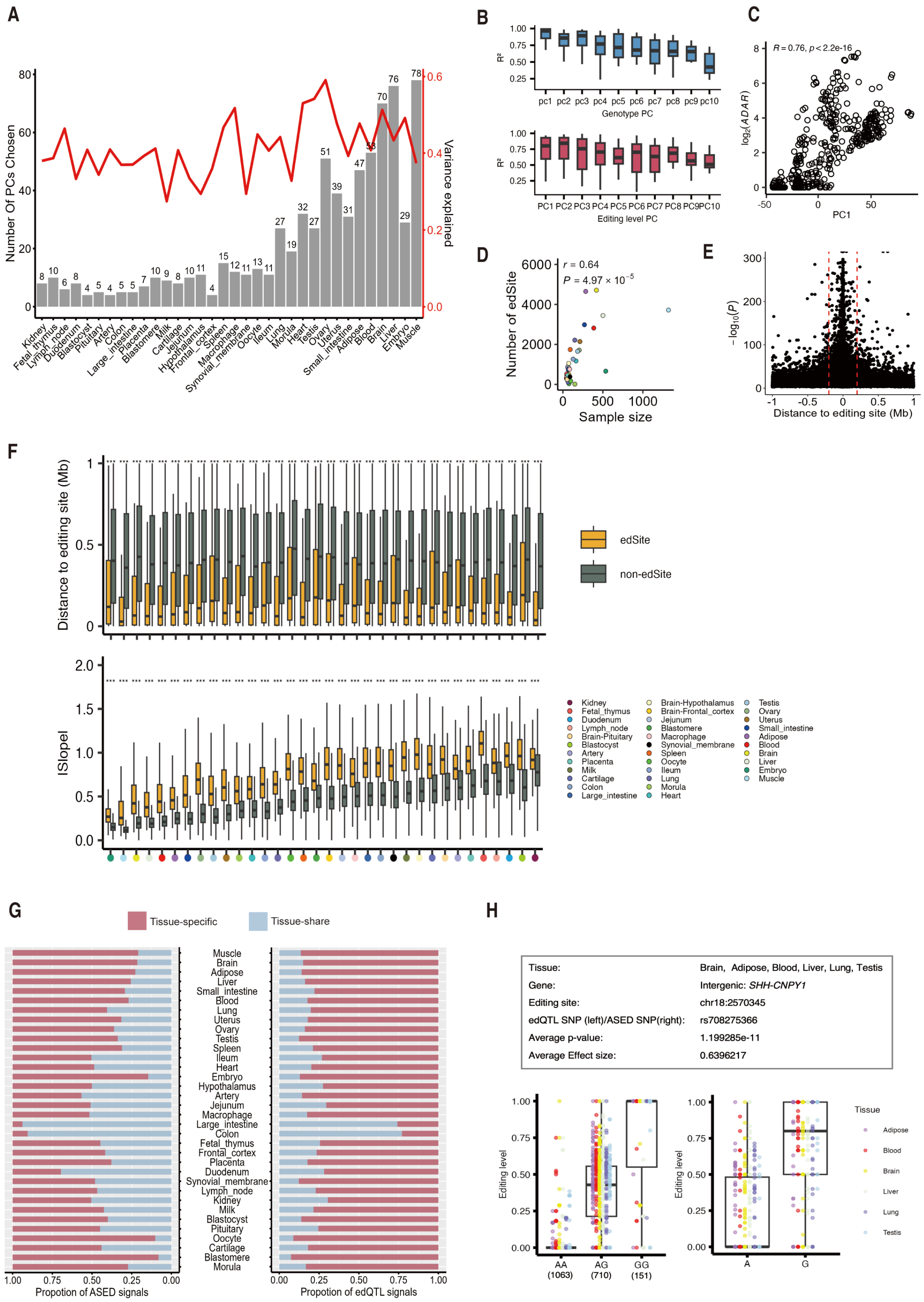

Fig. S5. Identification of cis-regulated RNA editing.

(A) Number of principal components (PCs) in each tissue. **(B)** Explained variance (R^2) of breed identity on the top ten genotype (left) and editing-level (right) principal components. **(C)** Association between *ADAR* expression level and PC1 in embryo samples (n = 536). **(D)** Pearson's

r between the number of detectable edSites and sample size across 34 tissues. **(E)** edQTL SNPs are highly enriched within 200 kb of their respective edSites. **(F)** The effect size (slope) of SNPs associated with editing sites (edQTLs) is significantly higher than that of SNPs near non-editing sites, whereas the physical distance between cis-edQTLs and their target edSites is significantly shorter, $^{*}P < 0.05$, $^{**}P < 0.01$ and $^{***}P < 0.001$. **(G)** Proportion of ASED (left) and edQTLs (right) in multiple tissues. **(H)** Example of an edQTL site (occurred in multiple tissues) in the intergenic of *SHH* and *CNPY1*.

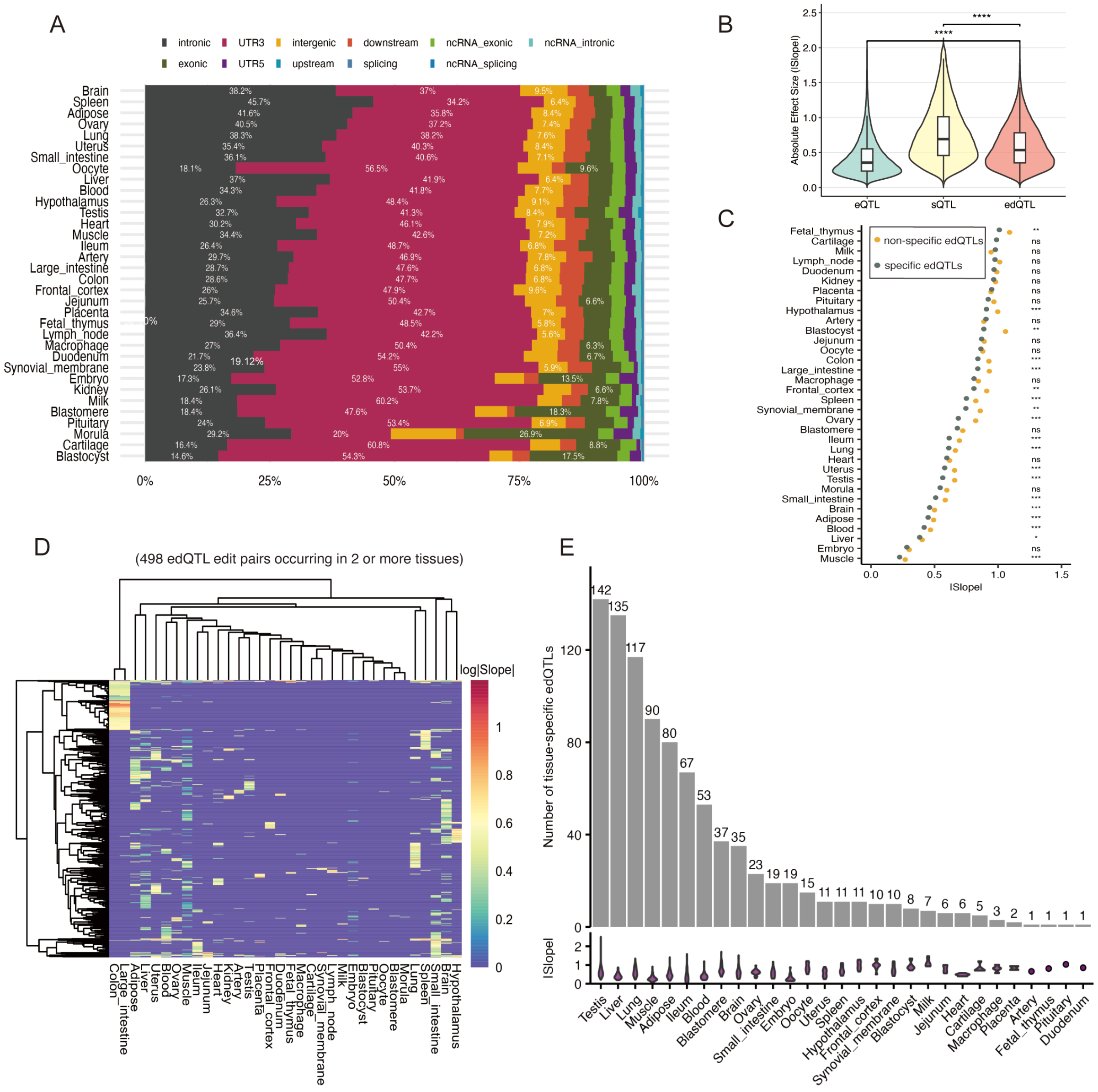

Fig. S6. Annotation of edSites and edQTLs in bulk tissue.

(A) Distribution characteristics of edSites genomic regions in different tissues, with only the top three proportion displayed in each tissue. **(B)** Comparisons of effect size across QTL types. **(C)**

Average effect values of specific and non-specific edQTLs in each tissue. **(D)** Heatmap of the slopes for 498 edQTL editing pairs with effects observed in multiple tissues. **(E)** Number of tissue-specific edQTLs identified in each tissue (top). The violin plots (bottom) show the distribution of absolute edQTL effect sizes (|slope|) in each corresponding tissue.

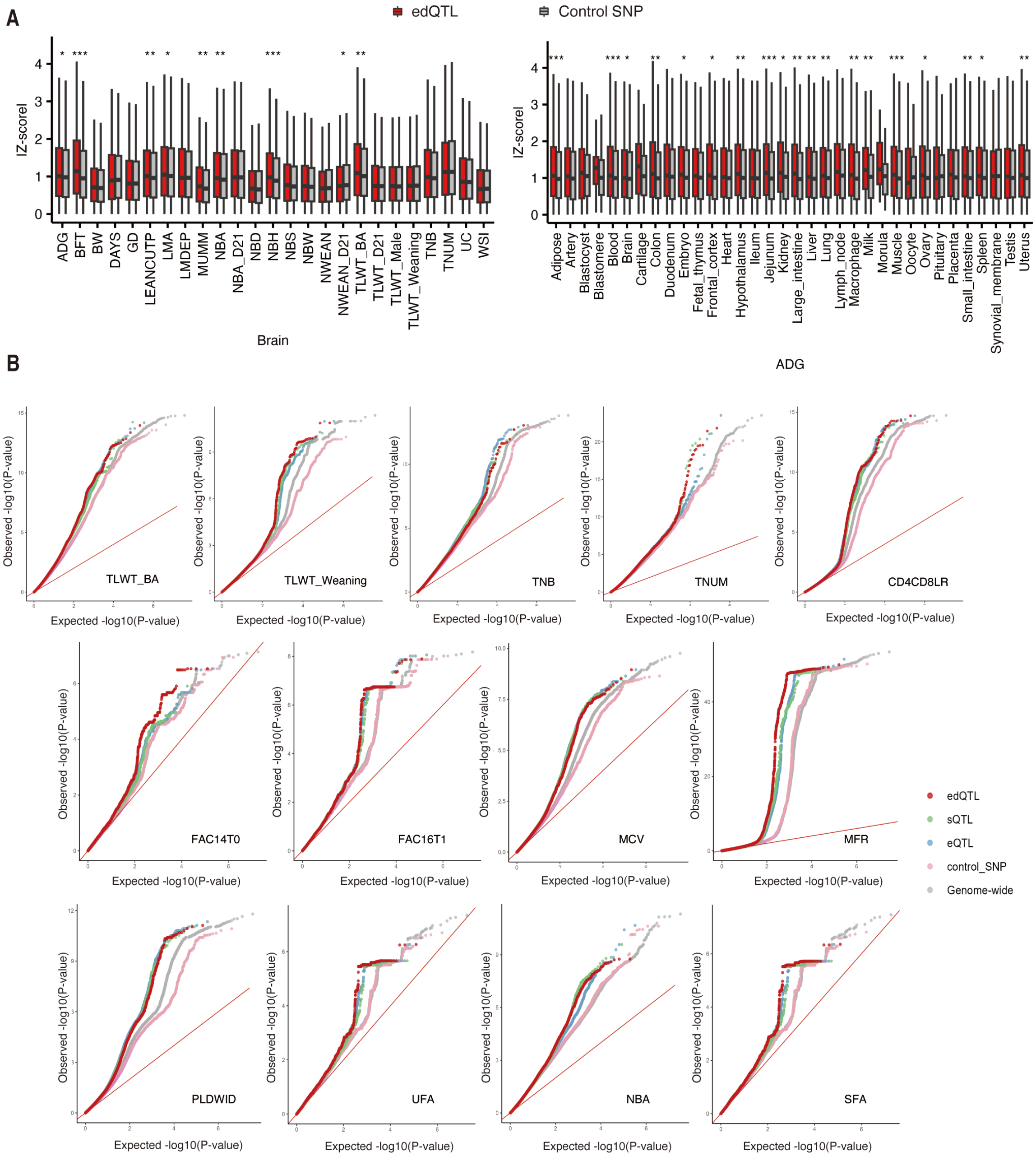

Fig. S7. The contribution of edQTLs to the phenotypic variation in complex traits.

(A) Z-score of edQTL and random SNPs for each trait in the brain(left), and z-score for each tissue in average daily gain (ADG) trait, $^{*}P < 0.05$, $^{**}P < 0.01$ and $^{***}P < 0.001$. **(B)** Quantile-quantile

plots of *P* values for GWAS SNPs marked as edQTLs, sQTLs and eQTLs, Random SNPs matching the edQTLs are used as negative controls.

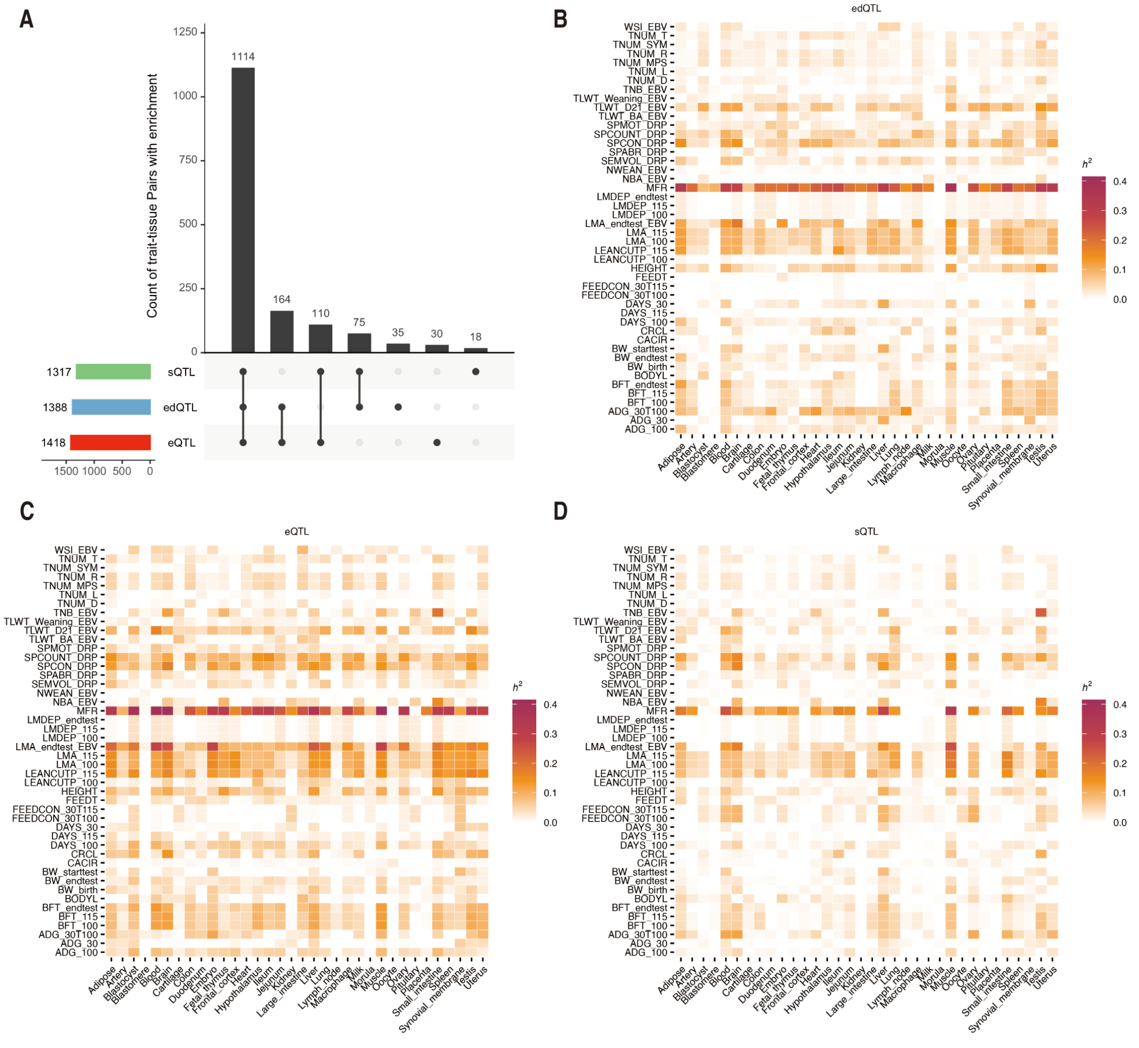

Fig. S8. Enrichment of different molecular QTLs in tissue-trait pairs.

(A) The number of heritability enrichments of edQTLs, eQTLs and sQTLs in tissue-trait pairs. **(B-D)** Heritability heatmaps of edQTL, eQTL and sQTL for tissue-trait pairs, respectively.

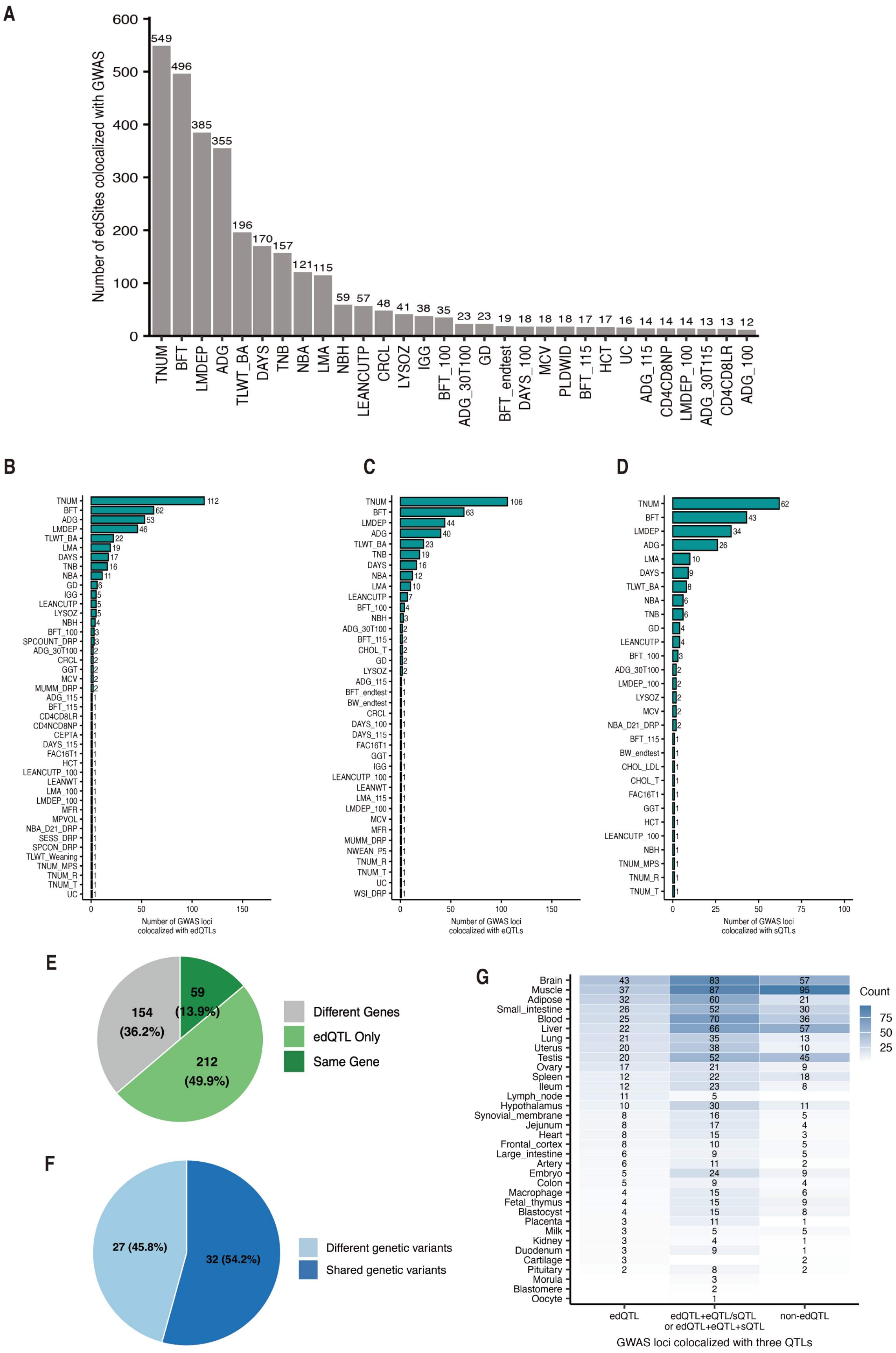
A
Number of edSites colocalized with GWAS
549 496 385 355 196 170 157 121 115 59 57 48 41 38 35 23 23 19 18 18 18 17 17 16 14 14 14 13 13 12
TNUM BFT LMDEP ADG TLWT_BA DAYS TNB NBA LMA NBH LEANCUTP CRCL LYSOZ IGG BFT_100 ADG_30T100 GD BFT_endtest DAYS_100 MCV PLDWID BFT_115 HCT UC ADG_115 CD4CD8NP LMDEP_100 ADG_30T115 CD4CD8LR ADG_100
B
TNUM 112, BFT 62, ADG 53, LMDEP 46, TLWT_BA 22, LMA 19, DAYS 17, TNB 16, NBA 11, GD 6, IGG 5, LEANCUTP 5, LYSOZ 5, NBH 4, BFT_100 3, SPCOUNT_DRP 3, ADG_30T100 2, CRCL 2, GGT 2, MCV 2, MUMM_DRP 2, ADG_115 1, BFT_115 1, CD4CD8LR 1, CD4NCD8NP 1, CEPTA 1, DAYS_115 1, FAC16T1 1, HCT 1, LEANCUTP_100 1, LEANWT 1, LMA_100 1, LMDEP_100 1, MFR 1, MPVOL 1, NBA_D21_DRP 1, SESS_DRP 1, SPCON_DRP 1, TLWT_Weaning 1, TNUM_MPS 1, TNUM_R 1, TNUM_T 1, UC 1
Number of GWAS loci colocalized with edQTLs
C
TNUM 106, BFT 63, LMDEP 44, ADG 40, TLWT_BA 23, TNB 19, DAYS 16, NBA 12, LMA 10, LEANCUTP 7, BFT_100 4, NBH 3, ADG_30T100 2, BFT_115 2, CHOL_T 2, GD 2, LYSOZ 2, ADG_115 1, BFT_endtest 1, BW_endtest 1, CRCL 1, DAYS_100 1, DAYS_115 1, FAC16T1 1, GGT 1, IGG 1, LEANCUTP_100 1, LEANWT 1, LMA_115 1, LMDEP_100 1, MCV 1, MFR 1, MUMM_DRP 1, NWEAN_P5 1, TNUM_R 1, TNUM_T 1, UC 1, WSI_DRP 1
Number of GWAS loci colocalized with eQTLs
D
TNUM 62, BFT 43, LMDEP 34, ADG 26, LMA 10, DAYS 9, TLWT_BA 8, NBA 6, TNB 6, GD 4, LEANCUTP 4, BFT_100 3, ADG_30T100 2, LMDEP_100 2, LYSOZ 2, MCV 2, NBA_D21_DRP 2, BFT_115 1, BW_endtest 1, CHOL_LDL 1, CHOL_T 1, FAC16T1 1, GGT 1, HCT 1, LEANCUTP_100 1, NBH 1, TNUM_MPS 1, TNUM_R 1, TNUM_T 1
Number of GWAS loci colocalized with sQTLs
E
154 (36.2%)
59 (13.9%)
212 (49.9%)
Different Genes
edQTL Only
Same Gene
F
27 (45.8%)
32 (54.2%)
Different genetic variants
Shared genetic variants
G
Count
edQTL
edQTL+eQTL/sQTL or edQTL+eQTL+sQTL
non-edQTL
GWAS loci colocalized with three QTLs
Brain 43 83 57
Muscle 37 87 95
Adipose 32 60 21
Small_intestine 26 52 30
Blood 25 70 36
Liver 22 66 57
Lung 21 35 13
Uterus 20 38 10
Testis 20 52 45
Ovary 17 21 9
Spleen 12 22 18
Ileum 12 23 8
Lymph_node 11 5
Hypothalamus 10 30 11
Synovial_membrane 8 16 5
Jejunum 8 17 4
Heart 8 15 3
Frontal_cortex 8 10 5
Large_intestine 6 9 5
Artery 6 11 2
Embryo 5 24 9
Colon 5 9 4
Macrophage 4 15 6
Fetal_thymus 4 15 9
Blastocyst 4 15 8
Placenta 3 11 1
Milk 3 5 5
Kidney 3 4 1
Duodenum 3 9 1
Cartilage 3 2
Pituitary 2 8 2
Morula 3
Blastomere 2
Oocyte 1

Fig. S9. Examining the overlap between edQTL, eQTL or sQTL and GWAS signals.

(A) Colocalization events of edQTL and GWAS signals. **(B-D)** Number of GWAS loci explained by edQTL, eQTL and sQTL in traits, respectively. **(E)** Concordance of target genes across all 425 edQTL–GWAS colocalized loci. **(F)** Concordance of genetic variation in all 59 genes. **(G)** The number of GWAS loci colocalized with non-edQTLs, edQTLs combined with eQTLs and/or sQTLs, and exclusive edQTLs across different tissues.

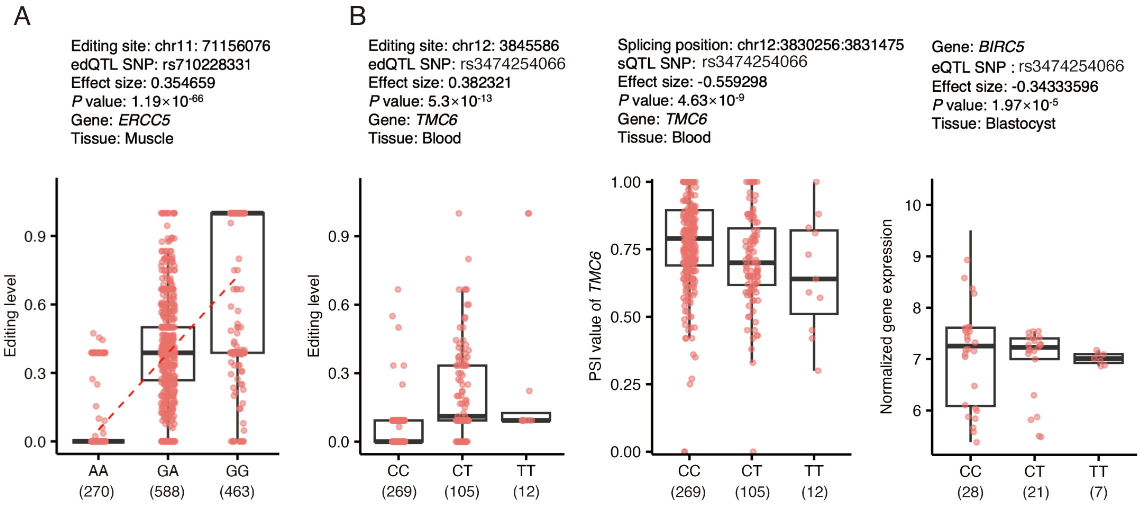

Fig. S10. Examples of the overlap between edQTL, eQTL or sQTL and GWAS signals.

(A) Box plots of the significant association of rs710228331 with the editing level at chr11: 71156076 in the muscle. **(B)** Box plots of the significant association of rs3474254066 with the editing level at chr12: 3845586 and alternative splicing values (PSI) of the *TMC6* gene within the blood, and the significant association of rs3474254066 with the expression level of the *BIRC5* gene in the blastocyst.

.
